# Supplementary material for: Small-molecule-mediated OGG1 inhibition attenuates pulmonary inflammation and lung fibrosis in a murine lung fibrosis model
Source: Nat Commun. 2023 Feb 6;14:643. doi: 10.1038/s41467-023-36314-5 (PMC9902543; doi:10.1038/s41467-023-36314-5)
Supplement: Supplementary file 1 — Supplementary Information [file 41467_2023_36314_MOESM1_ESM.pdf]

Small-molecule-mediated OGG1 inhibition attenuates pulmonary inflammation and lung fibrosis in a murine lung fibrosis model

One Sentence Summary: TH5487, an OGG1 inhibitor, significantly decreases pulmonary fibrosis.

L. Tanner[1]*, A.B. Single[1], R.K.V Bhongir[1], M. Heusel[2], T. Mohanty[2], C.A.Q. Karlsson[2], L. Pan[3], C-M. Clausson[4], J. Bergwik[1], K. Wang[3], C.K. Andersson[5], R. M. Oommen[6], J.S. Erjefält[4], J. Malmström[2], O. Wallner[6], I. Boldogh[3], T. Helleday[6,7,8], C. Kalderén[6, 7], A. Egesten[1]

[1]Respiratory Medicine & Allergology, Department of Clinical Sciences Lund, Lund University and Skåne University Hospital, SE-221 84 Lund, Sweden.

[2]Division of Infection Medicine, Department of Clinical Sciences, Lund University, SE-221 84 Lund, Sweden

[3]Department of Microbiology and Immunology, University of Texas Medical Branch at Galveston, Galveston, TX77555, USA

[4]Division of Airway Inflammation, Department of Experimental Medical Sciences, Lund University, SE-221 84 Lund, Sweden

[5]Respiratory Cell Biology, Department of Experimental Medical Sciences Lund, Lund University, SE-221 84 Lund, Sweden

[6]Science for Life Laboratory, Department of Oncology-Pathology, Karolinska Institutet, SE-171 76 Stockholm, Sweden.

[7]Oxcia AB, Norrbackagatan 70C, SE-113 34 Stockholm, Sweden.

[8]Weston Park Cancer Centre, Department of Oncology and Metabolism, University of Sheffield, Sheffield S10 2RX, UK.

*Corresponding author. Email: lloyd.tanner@med.lu.se.

This supplementary file contains:

Supplementary Figures 1-26

Supplementary Table 1 and 2

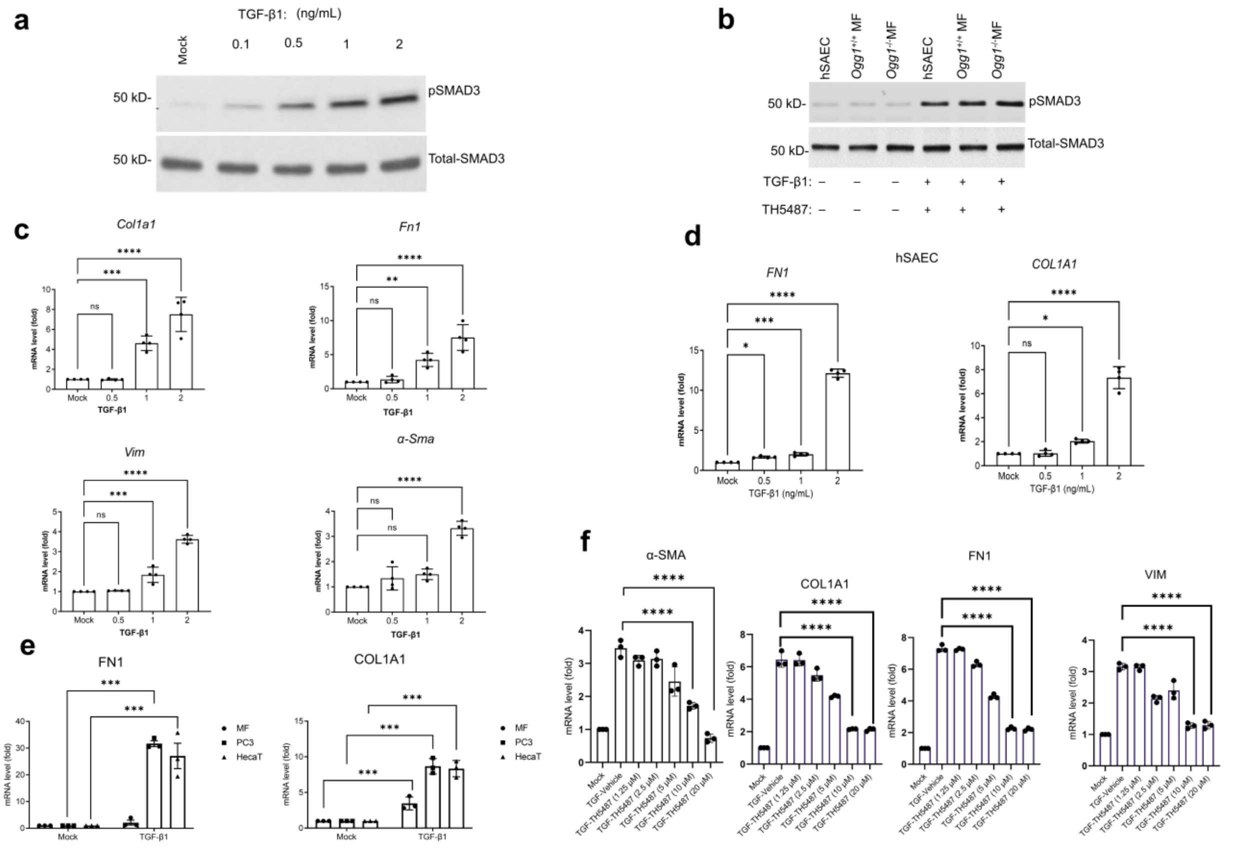

Supplementary Figure 1: TGF-β1-induced fibrotic gene expression is inhibited by TH5487. **A, B)** TGF-β1-induced phosphorylation of SMAD3 is not affected by lack of OGG1 or by the OGG1 inhibitor, TH5487. Parallel cultures of *Ogg1*$^{-/-}$ and *Ogg1*$^{+/+}$ MF cells were grown to ~70% confluence, kept in 0.5% FBS-containing medium for 24h and harvested at 1h after TGF-b1 [(0, 01, 0.5, 1 or 2 (A) and 2 ng/mL (B)] addition. Cell lysates were subjected to Western immunoblot analysis (n=3). **C, D)** TGF-β1 induced expression at mRNA levels of *αSma*, *Col1a1*, *Fn1* and *Vim* in *Ogg1*$^{+/+}$ MF. Cells (MF, hSAEC) were grown to ~70% confluence, starved as above, and were exposed to increasing concentrations of (0, 0.5, 1 or 2 ng/ml) TGF-β1. Total RNAs were isolated at 24h. Changes in mRNA levels were determined by qRT-PCR. Data were analyzed using one-way ANOVA with Dunnett's post-hoc test; Mock vs 2 ng/mL

TGF- β1 (*P*<0.0001; n=4) **E)** TGF-β1-induced expression of FN1 and COL1A1 in *Ogg1*$^{+/+}$ MF, PC3 and HaCat cells. Parallel cultures of cells ~70% confluency starved as in legend to C and D and mock- or TGF-β1-treated with 2 ng/mL. Changes in mRNA levels were determined by qRT-PCR. Data are representative of 3 independent experiments and were analyzed using a Student's t-test (two-sided): *FN1* Mock vs TGF-β1: MF (*P*=0.282); PC3 (*P*<0.0001); HaCat (*P*=0.005) and *COL1A1* Mock vs TGF-β1: MF (*P*=0.009); PC3 (*P*=0.0002); HaCat (*P*=0.0003) **F)** Dose-dependent inhibition by the OGG1 inhibitor, TH5487 of TGF-β1 induced gene expression. *Ogg1*$^{+/+}$ MF cells at 70% confluence were starved as in legend to **C-E** and increasing concentration (0, 1.25, 2.5, 5 and 10 μM) of TH5487 added 1h prior to TGF-b1 (2 ng/mL) addition for 24h. mRNA level of *αSma*, *Col1a1*, *Fn1* and *Vim* were determined by qRT-PCR. Single experiment performed with 3 parallel replicates. Statistical comparisons were conducted using Student's t-test (two-sided). ***P*<0.01, ****P*<0.005; *****P*<0.0001.

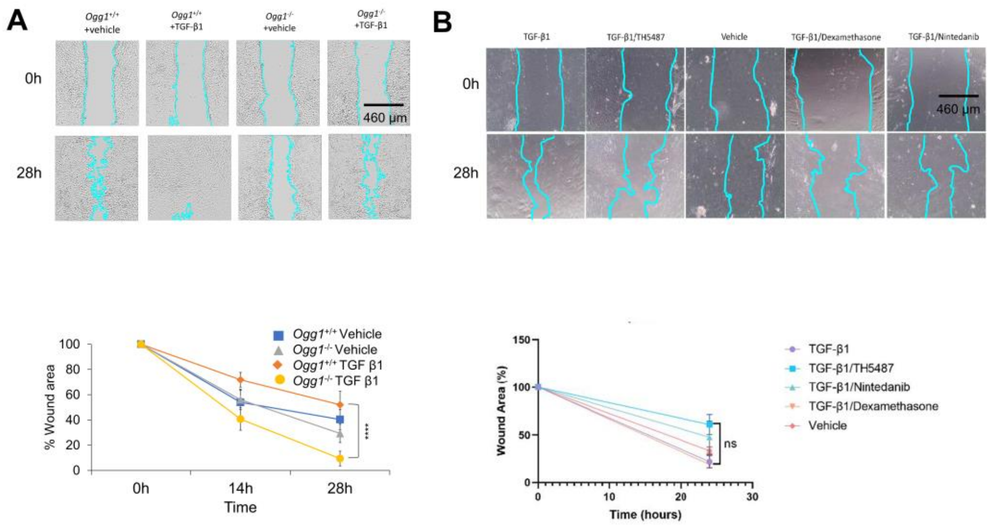

Supplementary Figure 2: TGF-β1-induced directional migration of *Ogg1*$^{+/+}$ and *Ogg1*$^{-/-}$ MF and pHLF cells as determined by wound healing assays. Following scratches, monolayers were washed and then mock-treated or 2 ng/mL TGF-β1 added ± inhibitor(s), TH5487 (10 µM) and Nintedanib (10 µM). Wound sizes were photographed at 0, 14, 28h. Data are presented as percentages of the initial wound area. Data are presented as means ± SEM of 3 independent experiments. Data are presented as means ±SEM (**A, B**). *Ogg1*$^{-/-}$ vs *Ogg1*$^{+/+}$ TGF-β1-treated cells showed significant differences, as determined using Student's t-test (P<0.0001) with no statistical difference reported for pHLF cells treated with TH5487 vs TGF-β1-treated cells (P=0.072).

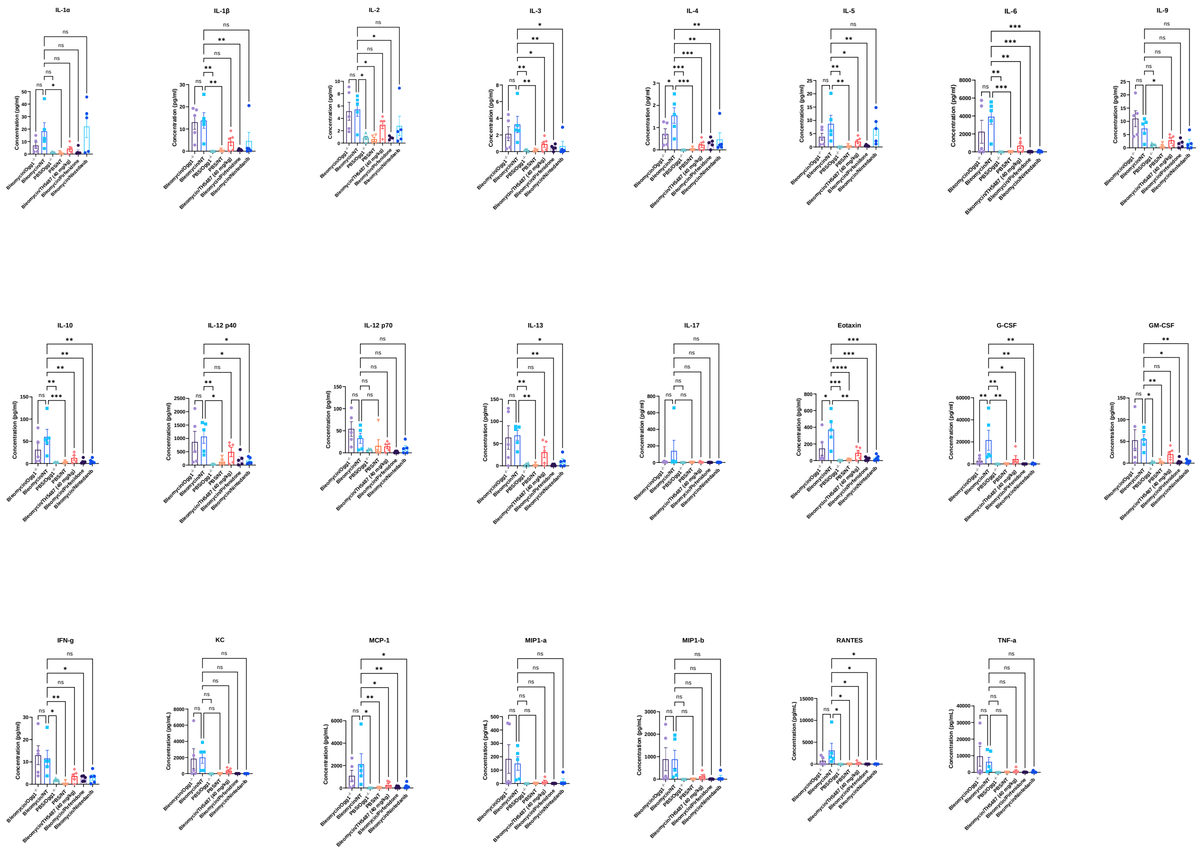

Supplementary Figure 3: Murine BALF cytokine levels from siRNA experiments. Cytokine levels were compared to the vehicle/bleomycin group using a one-way ANOVA (**P*<0.05; ***P*<0.01; ****P*<0.005; ******P*<0.001, and not significant-ns; Bleo/Ogg1 siRNA *n* = 5, Bleo/NT *n* = 5, PBS/Ogg1 siRNA *n*=*5*, PBS/NT *n* =5, Bleo/TH5487 *n* = 5, Bleo/Pirfenidone *n* =5, Bleo/Nintedanib *n* = 5). Data are presented as means ±SEM from 3 independent experiments.

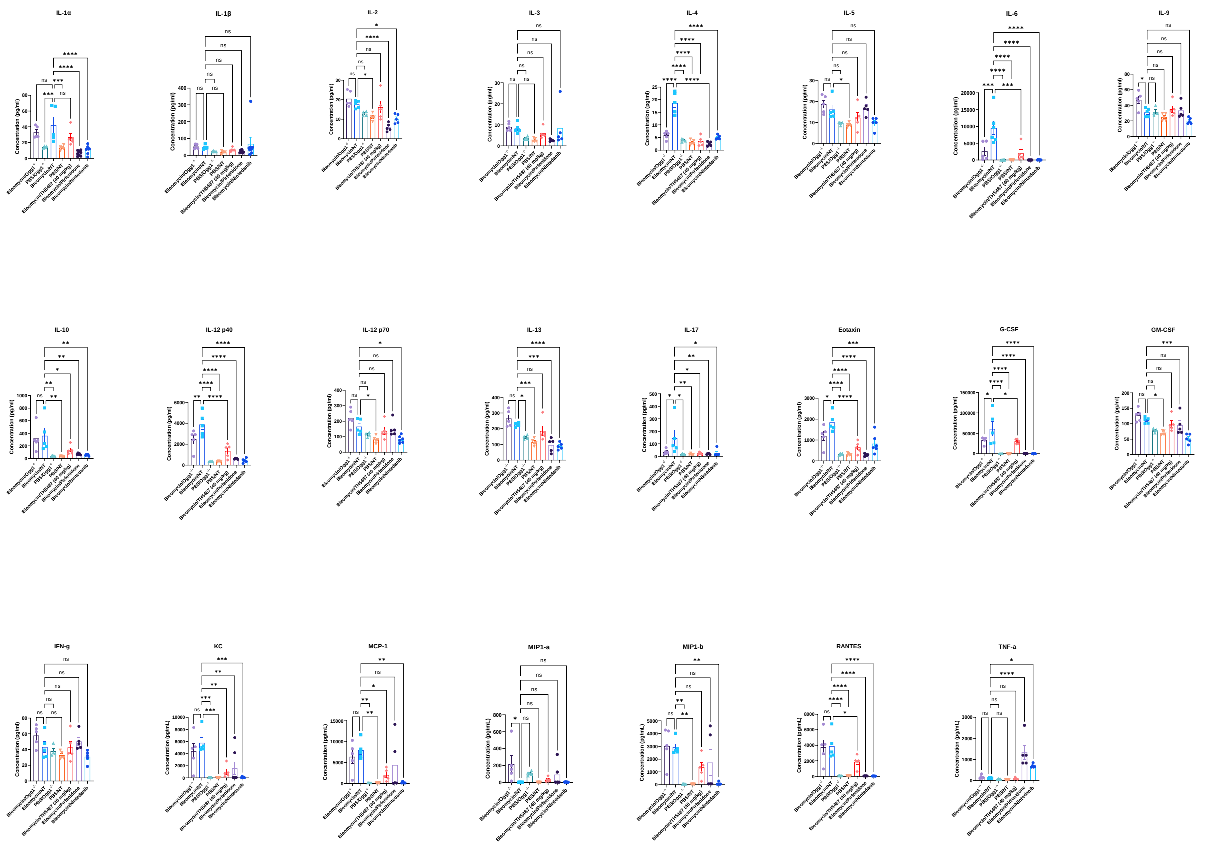

Supplementary Figure 4: Murine plasma cytokine levels from siRNA experiments. Cytokine levels were compared to the vehicle/bleomycin group using a one-way ANOVA (**P*<0.05; ***P*<0.01; ****P*<0.005; ******P*<0.001; Bleo/Ogg1 siRNA *n* = 5, Bleo/NT *n* = 5,

PBS/Ogg1 siRNA *n=5*, PBS/NT *n* =5, Bleo/TH5487 *n* = 5, Bleo/Pirfenidone *n* =5, Bleo/Nintedanib *n* = 5). Data are presented as means ±SEM from 3 independent experiments.

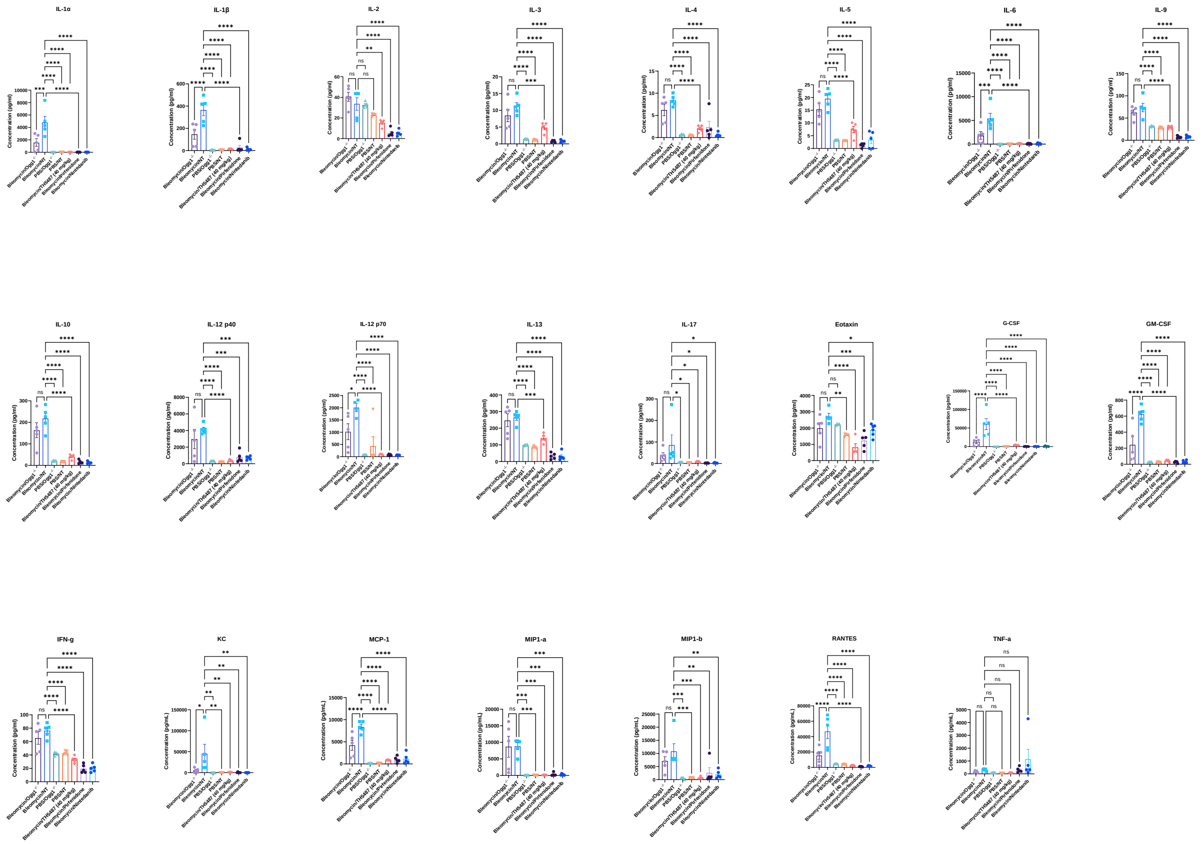

Supplementary Figure 5: Murine lung homogenate cytokine levels from siRNA experiments. Cytokine values were compared to the vehicle/bleomycin group using a one-way ANOVA (**P*<0.05*;* ***P*<0.01*;* ****P*<0.005*;* ******P*<0.001; Bleo/Ogg1 siRNA *n* = 5, Bleo/NT *n* = 5, PBS/Ogg1 siRNA *n=5*, PBS/NT *n* =5, Bleo/TH5487 *n* = 5, Bleo/Pirfenidone *n* =5, Bleo/Nintedanib *n* = 5). Data are presented as means ±SEM from 3 independent experiments.

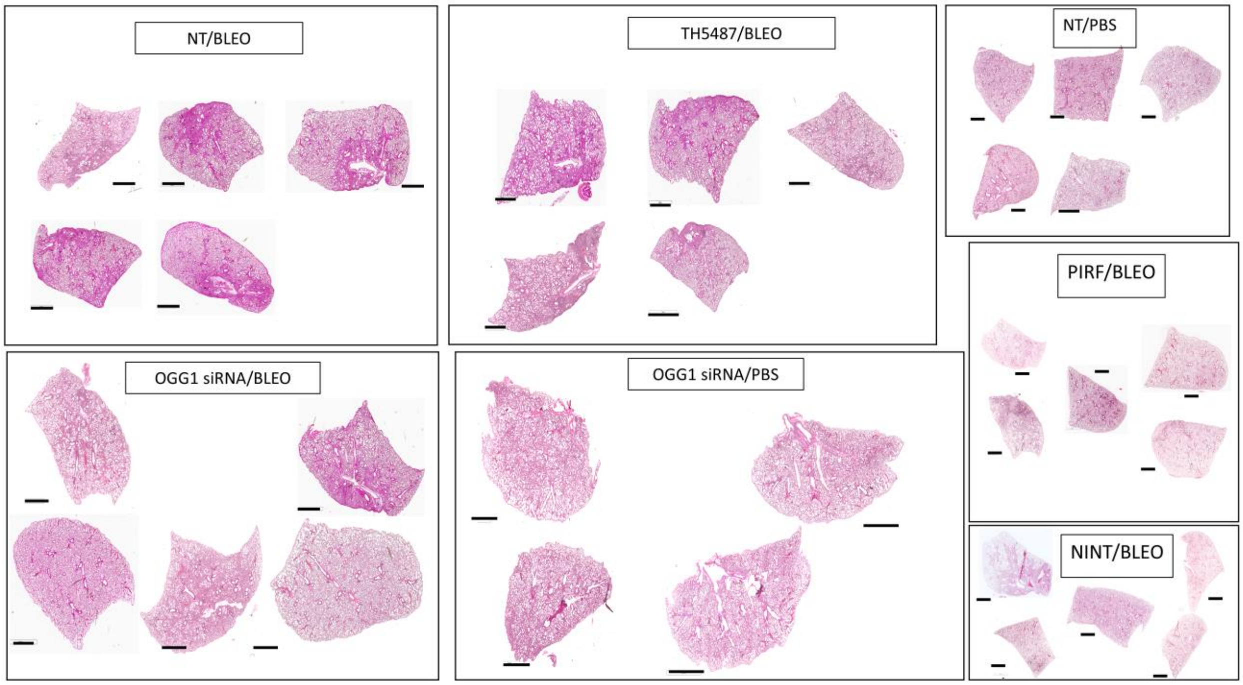

Supplementary Figure 6: Whole lung scans of murine lungs from siRNA experiments following H&E staining (scale bar=2 mm). NT- non-targeting siRNA; PBS- phosphate-buffered saline; PIRF-pirfenidone; NINT-nintedanib. Results shown from 3 independent experiments.

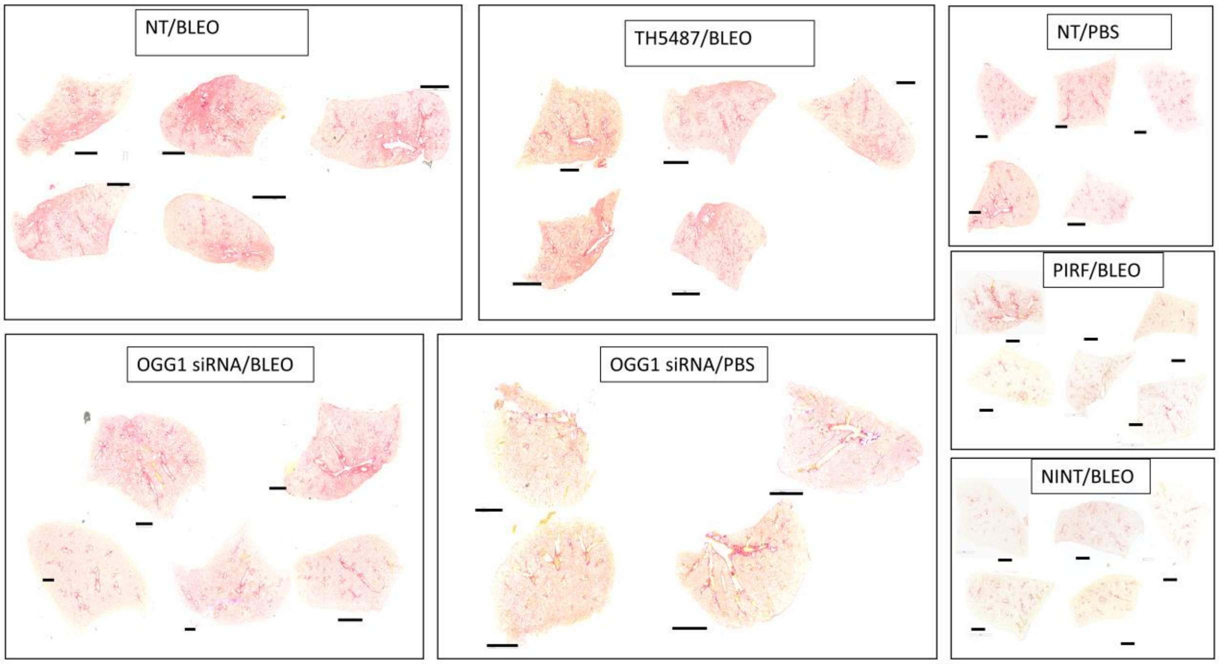

Supplementary Figure 7: Whole lung scans of murine lungs from siRNA experiments following picrosirius red staining (scale bar=2 mm). NT- non-targeting siRNA; PBS- phosphate- buffered saline; PIRF-pirfenidone; NINT-nintedanib. Results shown from 3 independent experiments.

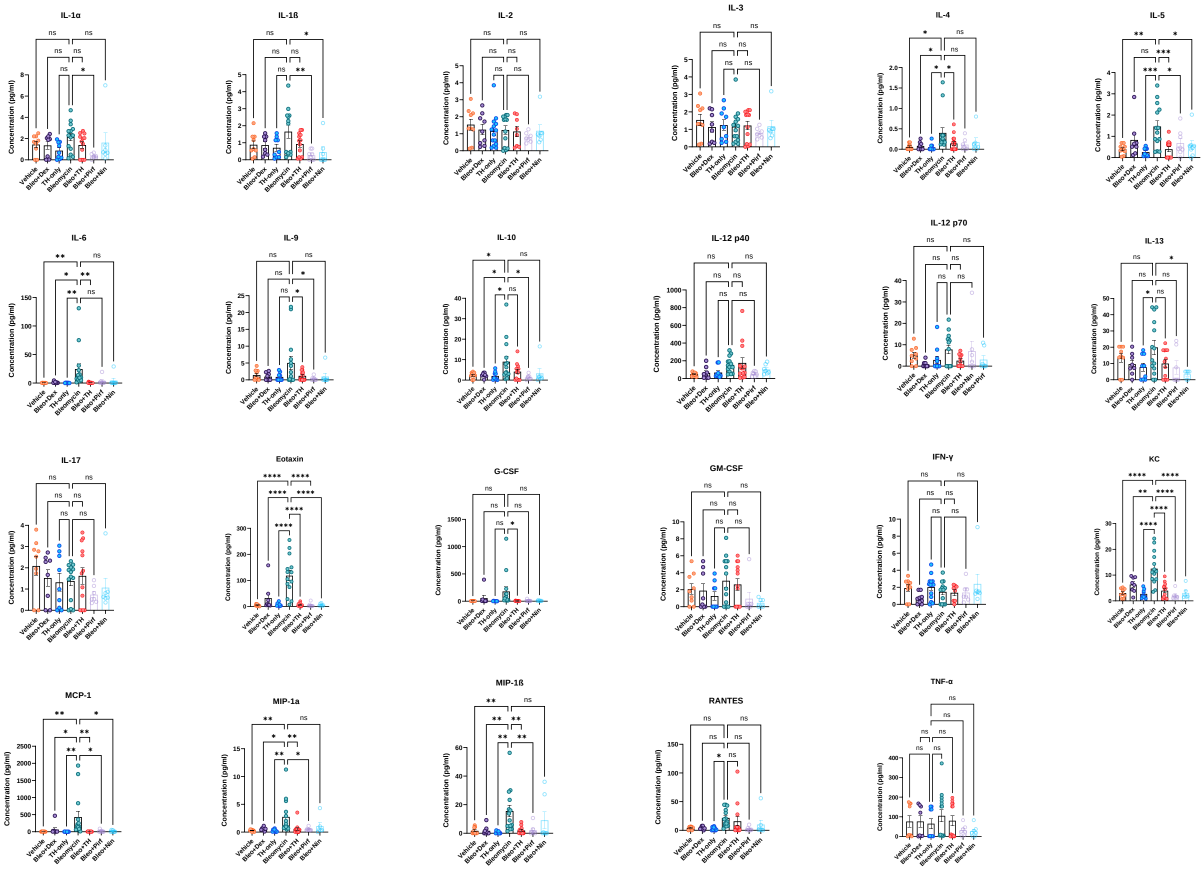

Supplementary Figure 8: Murine BALF cytokine levels. Cytokine values were compared to the vehicle/bleomycin group using a one-way ANOVA (**P*<0.05*;* ***P*<0.01*;* ****P*<0.005*;* ******P*<0.001, and not significant-ns; Vehicle *n* = 9, TH-only *n* = 9, Bleomycin *n*=14, Bleo+TH *n* =13, Bleo+Dex *n* = 8, Bleo+Pirf *n* = 7, Bleo/Nintedanib *n* = 7). Data are presented as means ± SEM from 2 independent experiments.

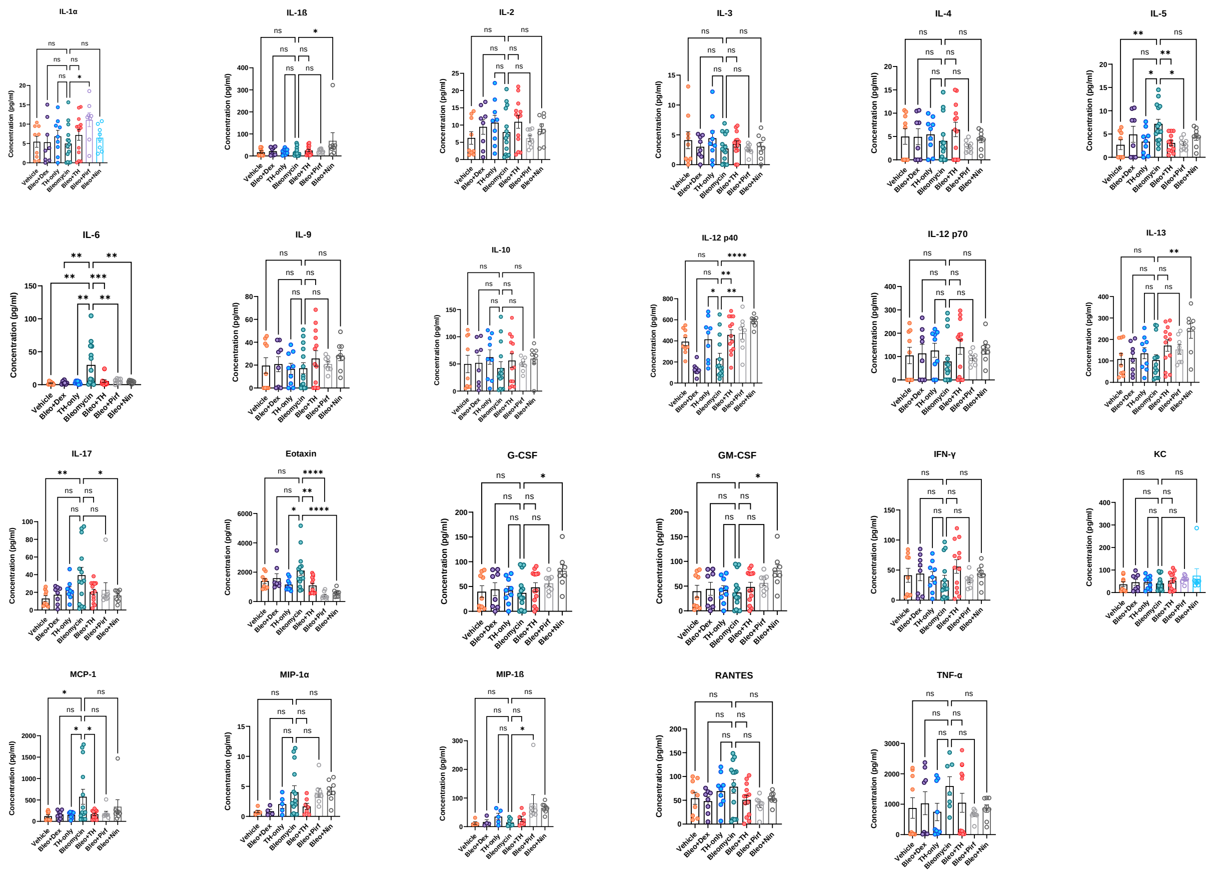

Supplementary Figure 9: Murine plasma cytokine levels. Cytokine values were compared to the vehicle/bleomycin group using a one-way ANOVA (**P*<0.05*;* ***P*<0.01*;* ******P*<0.001, and not significant-ns). Vehicle *n* = 9, TH-only *n* = 9, Bleomycin *n*=14, Bleo+TH *n* =13, Bleo+Dex *n* = 8, Bleo+Pirf *n* = 7, Bleo/Nintedanib *n* = 7). Data are presented as means ± SEM from 2 independent experiments.

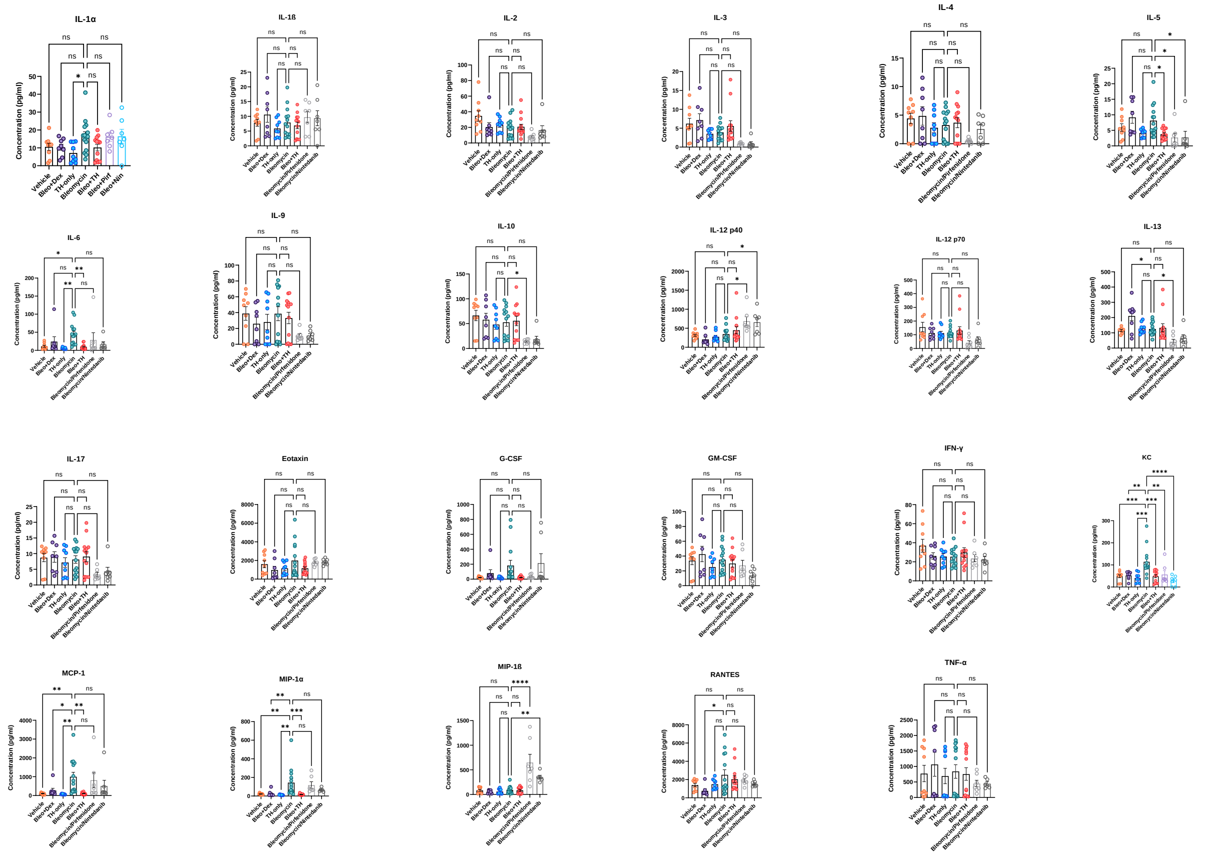

Supplementary Figure 10: Murine lung homogenate cytokine levels. Cytokine values were compared to the vehicle/bleomycin group using a one-way ANOVA (**P*<0.05*;* ***P*<0.01*;* ****P*<0.005*;* ******P*<0.001, and not significant-ns). Vehicle *n* = 9, TH-only *n* = 9, Bleomycin *n*=14, Bleo+TH *n* =13, Bleo+Dex *n* = 8, Bleo+Pirf *n* = 7, Bleo/Nintedanib *n* = 7). Data are presented as means ± SEM from 2 independent experiments.

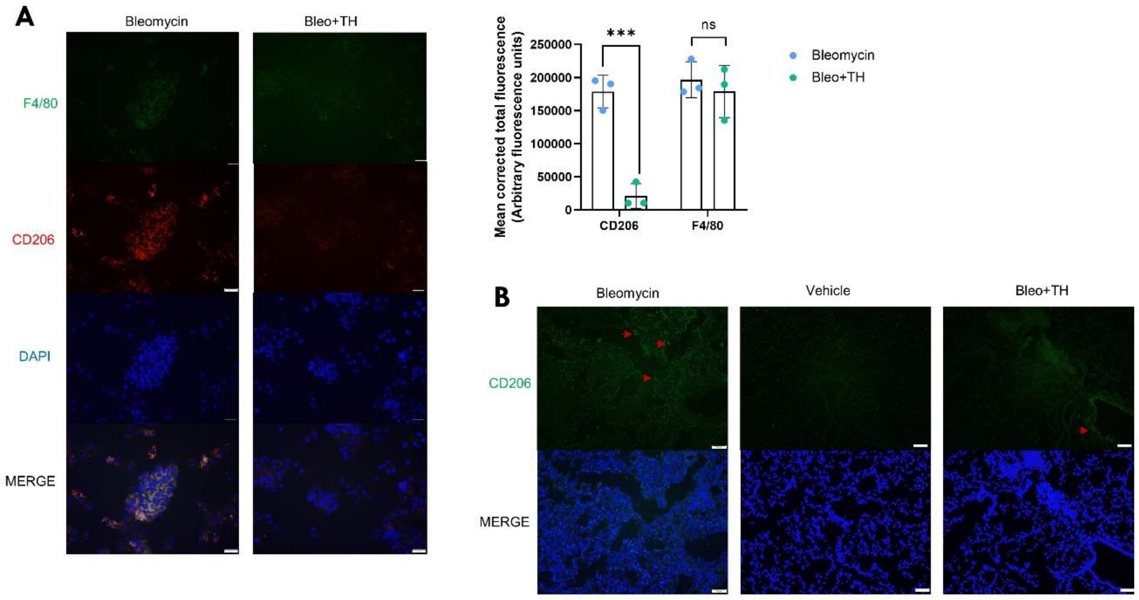

Supplementary Figure 11: Macrophage-specific staining in (A) mouse BALF and (B) lung sections following TH5487 (TH) treatment (prophylactic treatment). F4/80 (green), CD206 (red/green), and DAPI (blue) immunofluorescence staining, with quantification (n=3 samples) inset. Scale bar = 50 µm. Data are presented as means ±SEM. CD206 staining showed a significant difference between Bleo vs Bleo/TH-treated samples (*P*=0.003) and no statistical significance when stained with F4/80 (*P* = 0.91). Statistical analysis was conducted using a Student’s t-test.

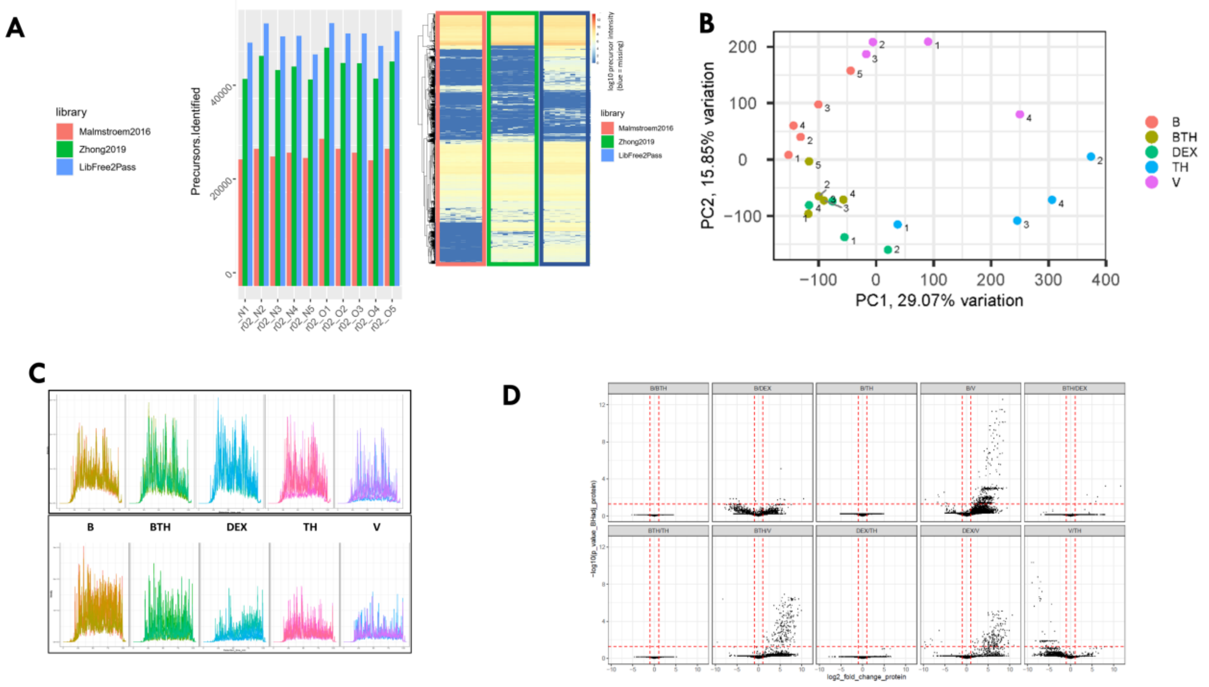

Supplementary Figure 12: Proteomic DIA analysis of mouse lung samples from 5 treatment conditions; bleomycin (B), bleomycin/TH5487 (BTH), bleomycin/dexamethasone (DEX), TH5487 only (TH), and vehicle (V). (A) Increased precursor identifications were found using LibFree2Pass methodology in this study compared with prior-knowledge-based spectral library approaches (B) PCA plot showing treatment condition groupings. (C) Representative chromatograms, (D) Volcano plot displaying condition comparisons in the lung samples analyzed. Cohort included Bleo (B) *n* = 5, bleomycin/TH5487 (BTH) *n* =5, bleomycin/dexamethasone (DEX) *n* = 4, TH5487 only (TH) *n* = 4 and vehicle only (V) *n* = 4, respectively.

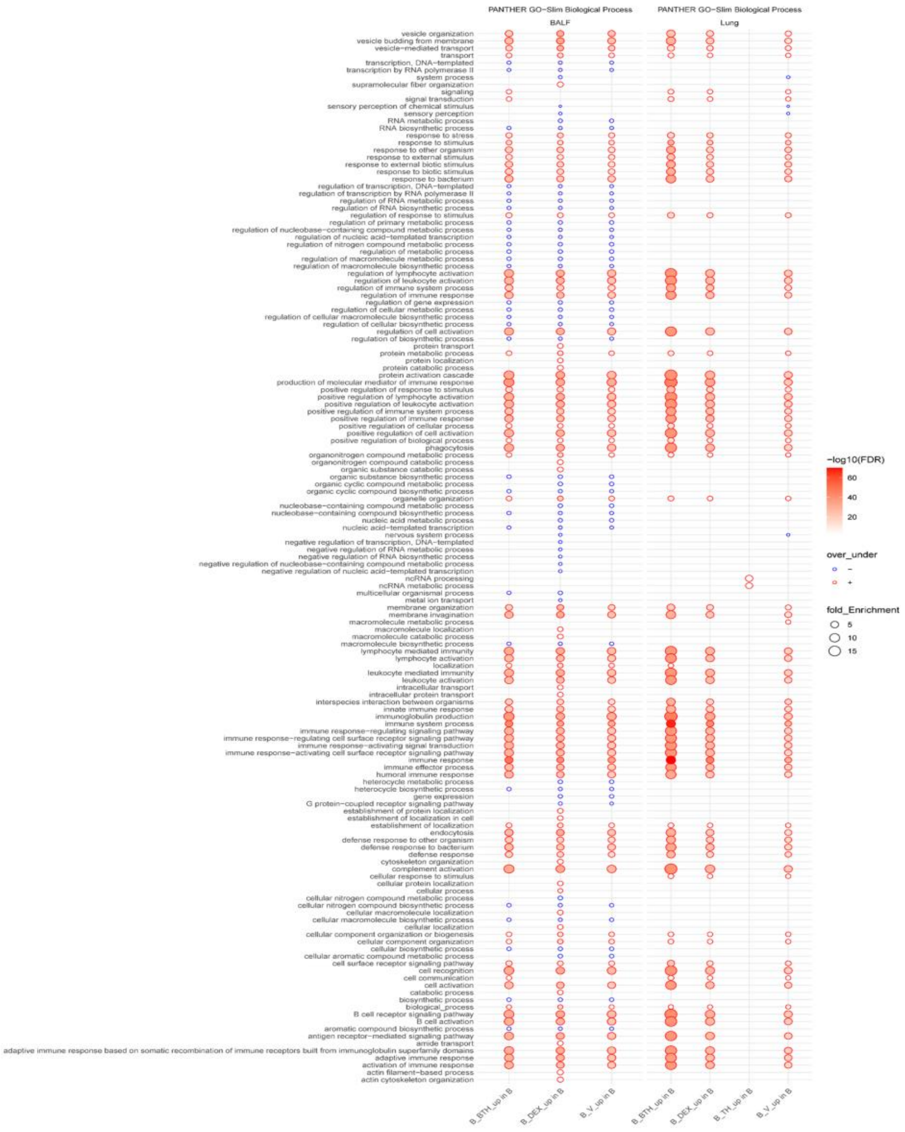

Supplementary Figure 13: PANTHER gene ontology (GO) biological process enrichment analysis from mouse BALF and lung homogenate protein list IDs. Top 100 biologically up/downregulated processes are presented here. Treatment conditions compared include

bleomycin (B), bleomycin/TH5487 (BTH), bleomycin/dexamethasone (DEX), TH5487 only (TH), and vehicle (V). FDR-false discovery rate. Cohort included Bleo (B) *n* = 5, bleomycin/TH5487 (BTH) *n* =5, bleomycin/dexamethasone (DEX) *n* = 4, TH5487 only (TH) *n* = 4 and vehicle only (V) *n* = 4, respectively.

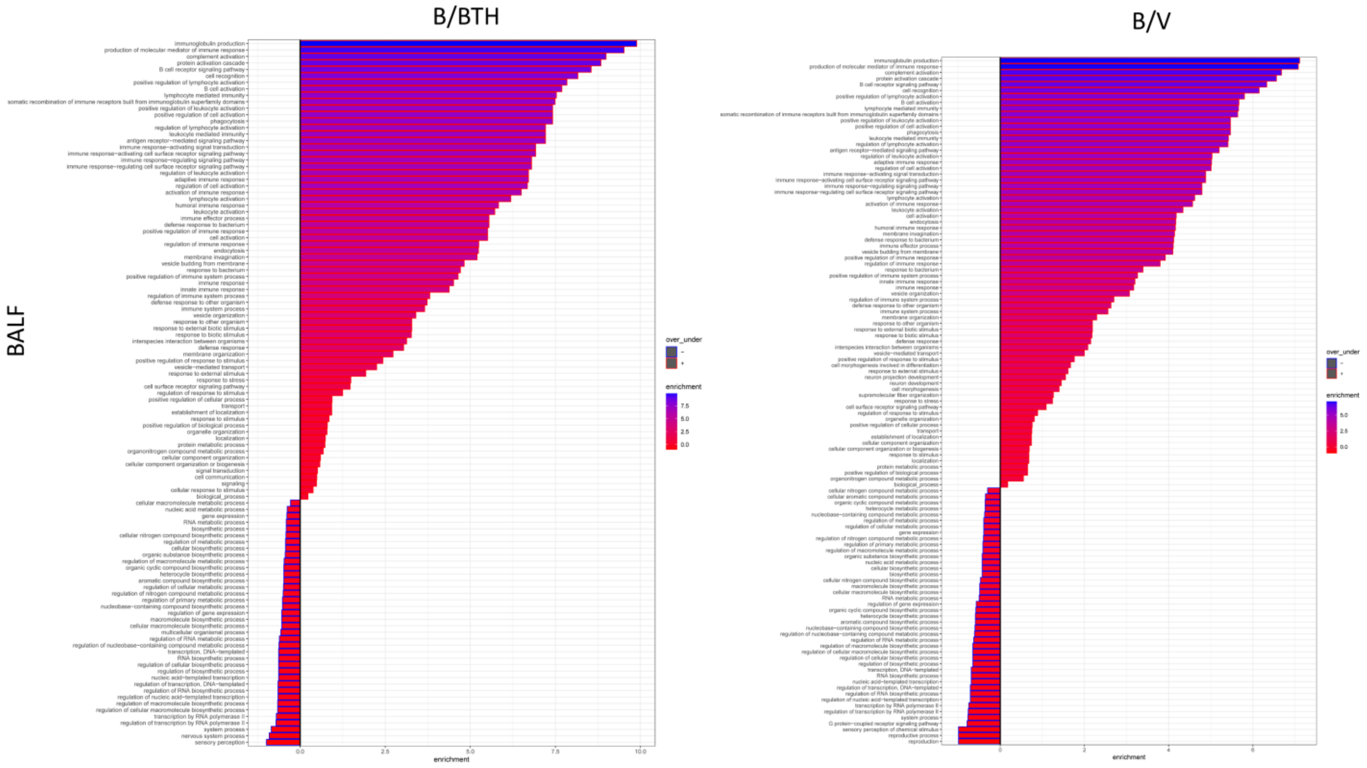

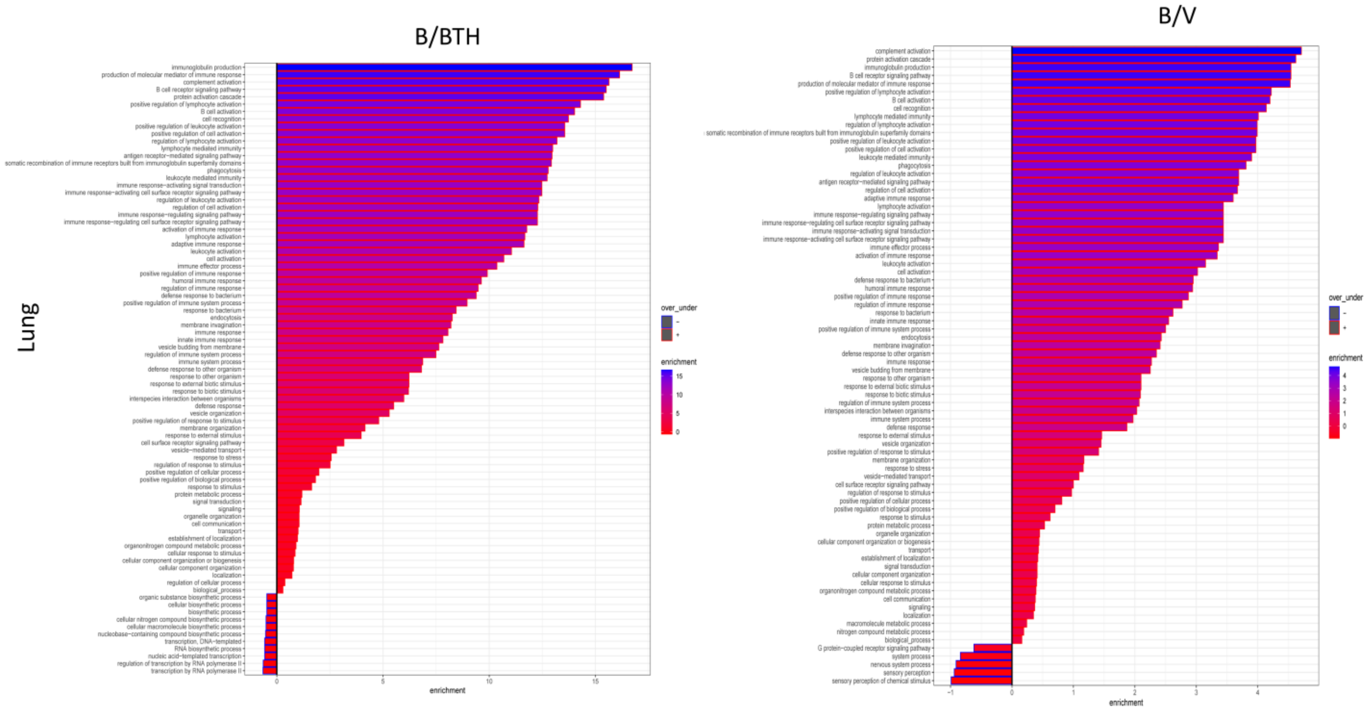

Supplementary Figure 14: PANTHER gene ontology (GO) slim biological process enrichment analysis from mouse BALF and lung homogenate protein list IDs comparing bleomycin/TH-treated and bleomycin/untreated samples. Bar graphs show the top 50 up/down regulated biological processes following each treatment, respectively. Blue-significantly up/down; red-less significantly up/down regulated. Cohort included Bleo (B) *n* = 5, bleomycin/TH5487 (BTH) *n* =5 and vehicle only (V) *n* = 4, respectively.

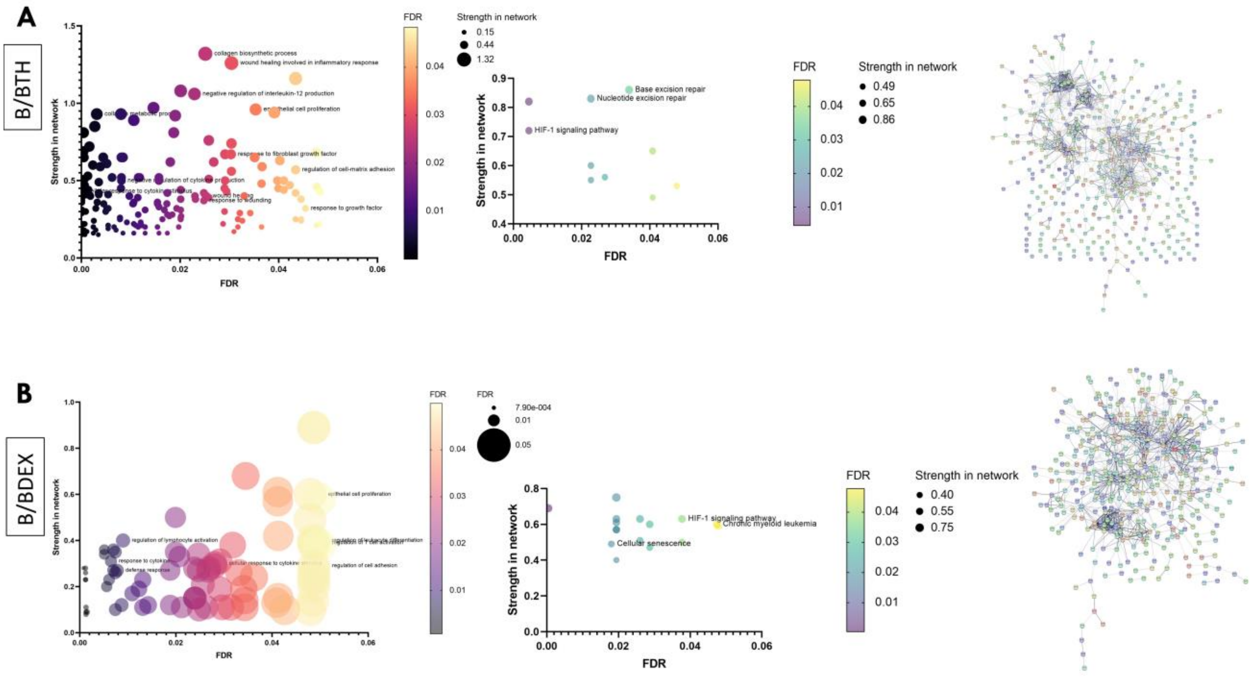

Supplementary Figure 15: STRINGDB gene ontology (GO; yellow/red/black figure) and Kyoto encyclopedia of genes and genomes (KEGG; yellow/green/purple figure) terms from lung homogenate samples. (A) Bleomycin (B) vs Bleo/TH5487 (BTH)-treated samples, (B) Bleomycin/dexamethasone (DEX) treatment conditions; false discovery rate (FDR). Cohort included Bleo (B) *n* = 5, bleomycin/TH5487 (BTH) *n* =5, bleomycin/dexamethasone (DEX) *n* = 4.

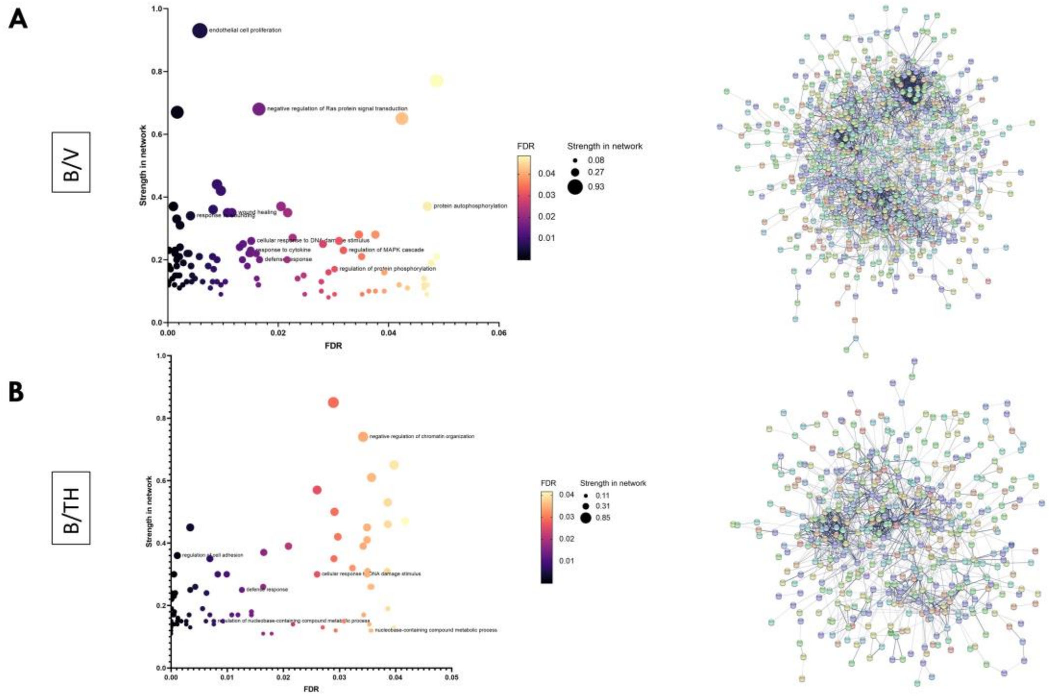

Supplementary Figure 16: STRINGDB gene ontology (GO) terms from mouse lung homogenate samples. (A) Bleomycin/vehicle (V), (B) Bleomycin/TH5487 only (TH) treatment conditions; false discovery rate (FDR). Cohort included Bleo (B) *n* = 5, bleomycin/TH5487 (BTH) *n* =5 and vehicle only (V) *n* = 4, respectively.

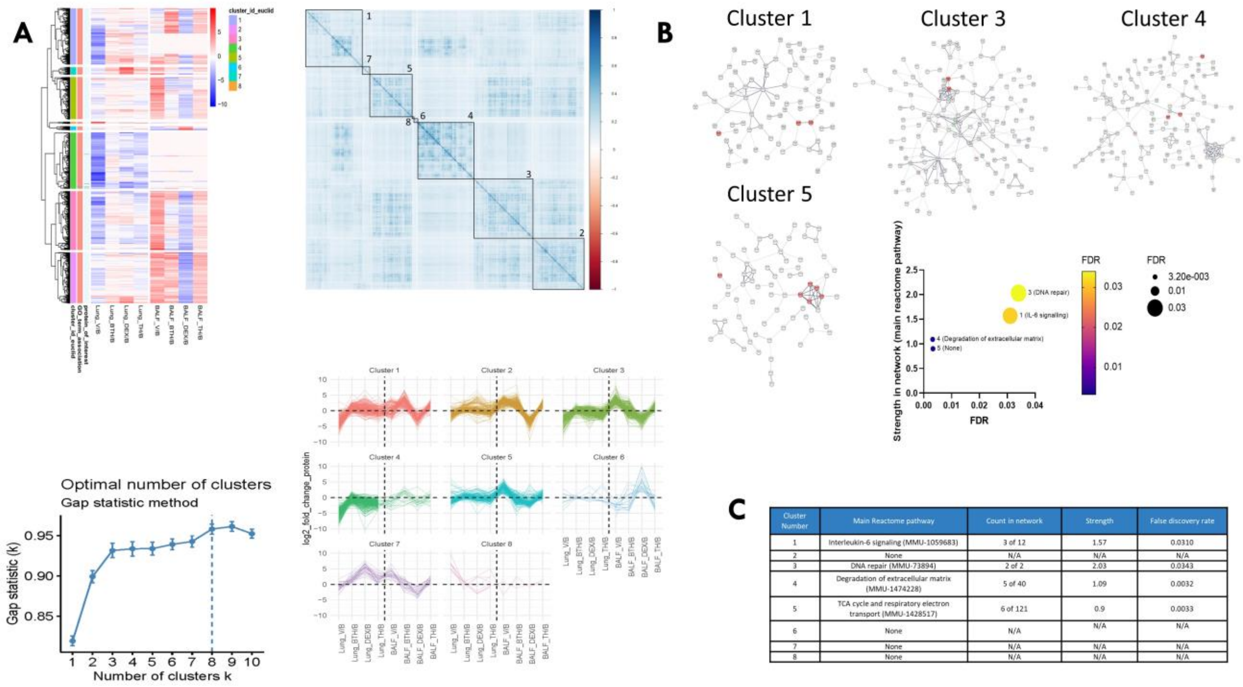

Cluster Number	Main Reactome pathway	Count in network	Strength	False discovery rate
1	Interleukin-6 signaling (MMU-1059683)	3 of 12	1.57	0.0310
2	None	N/A	N/A	N/A
3	DNA repair (MMU-73894)	2 of 2	2.03	0.0343
4	Degradation of extracellular matrix (MMU-1474228)	5 of 40	1.09	0.0032
5	TCA cycle and respiratory electron transport (MMU-1428517)	6 of 121	0.9	0.0033
6	None	N/A	N/A	N/A
7	None	N/A	N/A	N/A
8	None	N/A	N/A	N/A

Supplementary Figure 17: STRINGDB gene ontology (GO) terms from mouse lung homogenate samples analyzed using cluster analysis. (A) Differential cluster identification analysis identifying differentially expressed proteins in the lungs and BALF of relevant treatment comparisons, with optimal cluster number identified by the gap statistic method. Cohort included Bleo (B) *n* = 5, bleomycin/TH5487 (BTH) *n* =5, bleomycin/dexamethasone (DEX) *n* = 4, TH5487 only (TH) *n* = 4 and vehicle only (V) *n* = 4, respectively. Data are presented as means ±SEM. **(B)** Log2FC changes are depicted for each cluster with protein number and main reactome pathways identified in each. **(C)** Representative network analysis diagram displaying proteins from cluster 4, associated with extracellular matrix degradation. False discovery rate-FDR. Treatment conditions compared include bleomycin (B), bleomycin/TH5487 (BTH), bleomycin/dexamethasone (DEX), TH5487 only (TH), and vehicle (V). FDR-false discovery rate.

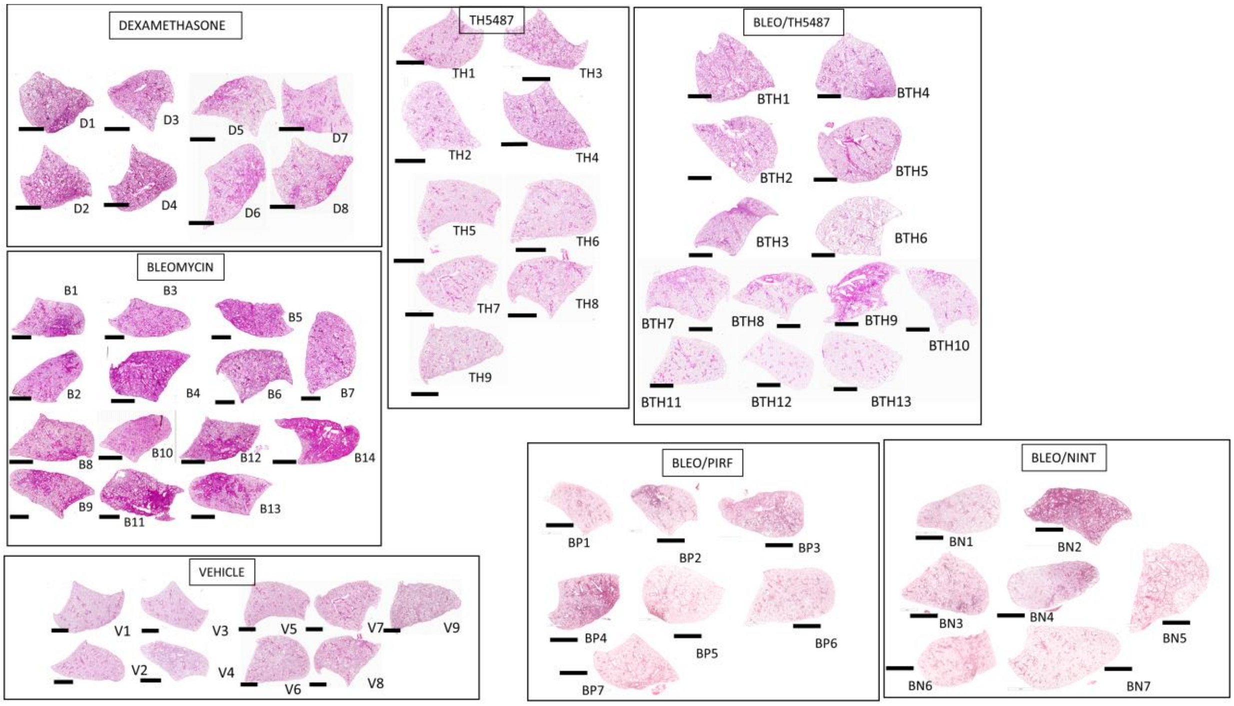

Supplementary Figure 18: Whole lung scans of murine lungs following H&E staining (scale bar=2 mm). Treatment conditions compared include bleomycin, bleomycin/TH5487 (Bleo/TH5487), bleomycin/dexamethasone (D), TH5487 only, vehicle, bleomycin/pirfenidone (Bleo/PIRF), and bleomycin/nintedanib (Bleo/NINT). Results shown from 2 independent experiments.

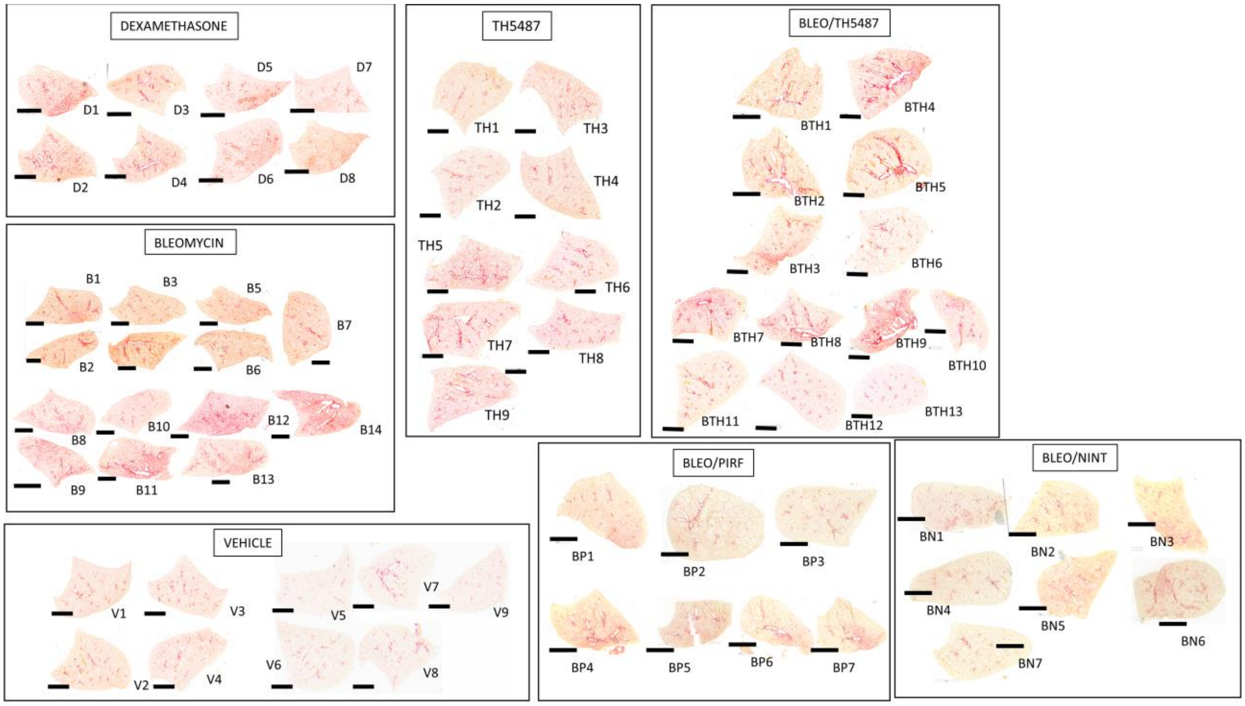

Supplementary Figure 19: Whole lung scans of murine lungs following picrosirius red staining (scale bar=2 mm). Treatment conditions compared include bleomycin, bleomycin/TH5487 (Bleo/TH5487), bleomycin/dexamethasone (D), TH5487 only, vehicle, bleomycin/pirfenidone (Bleo/PIRF), and bleomycin/nintedanib (Bleo/NINT). Results shown from 2 independent experiments.

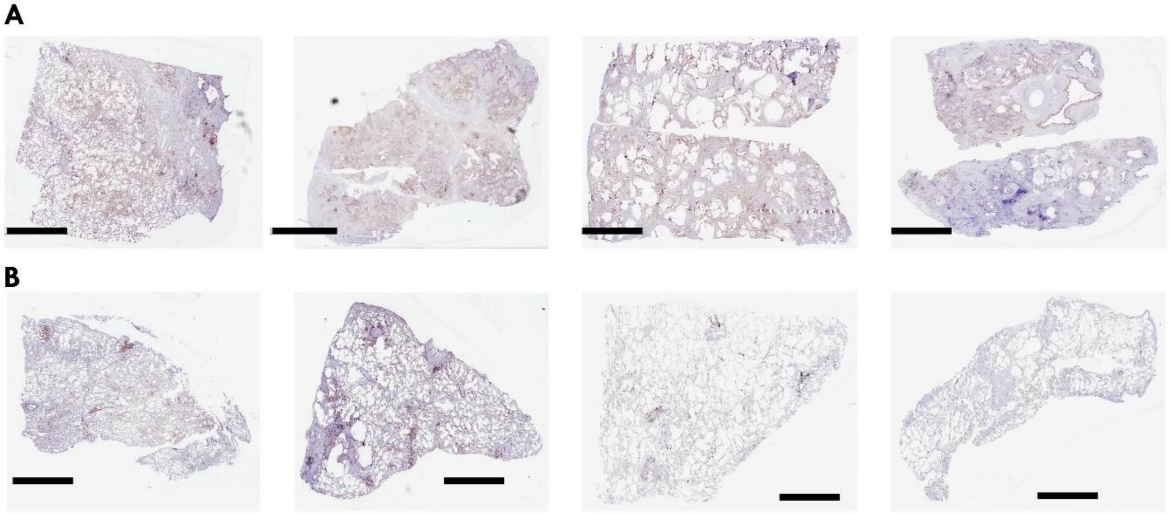

Supplementary Figure 20: Lung scans of human lung explants following immunostaining for OGG1. (A) IPF lung tissue (n=4) and (B) control tissue (n=4). Scale bar=6 mm.

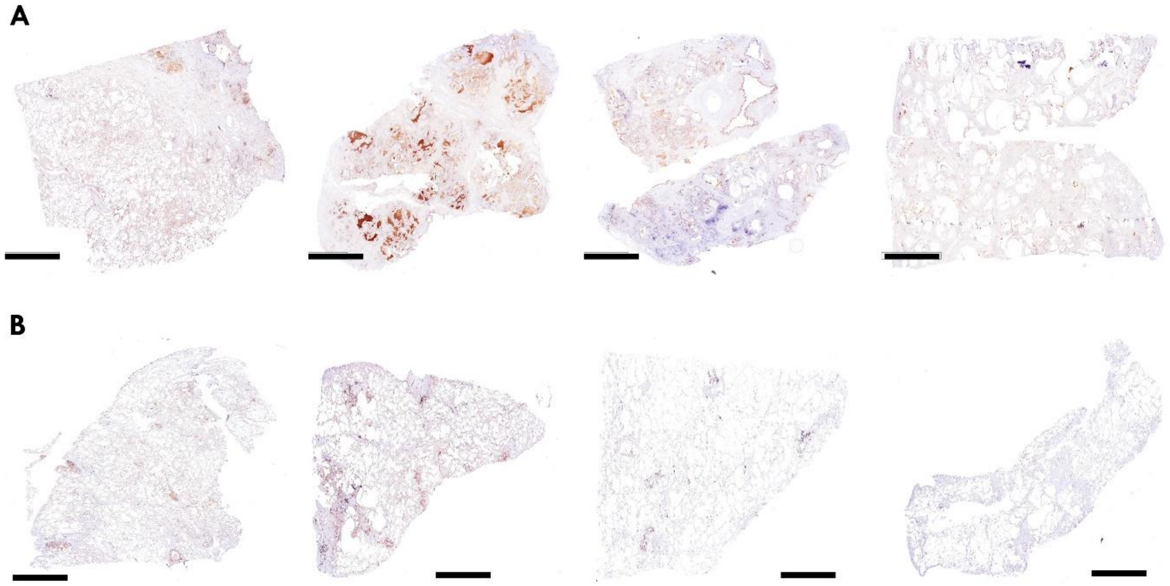

Supplementary Figure 21: Lung scans of human lung explants following immunostaining for SMAD7. (A) IPF lung tissue (n=4) and (B) control tissue (n=4). Scale bar=6 mm.

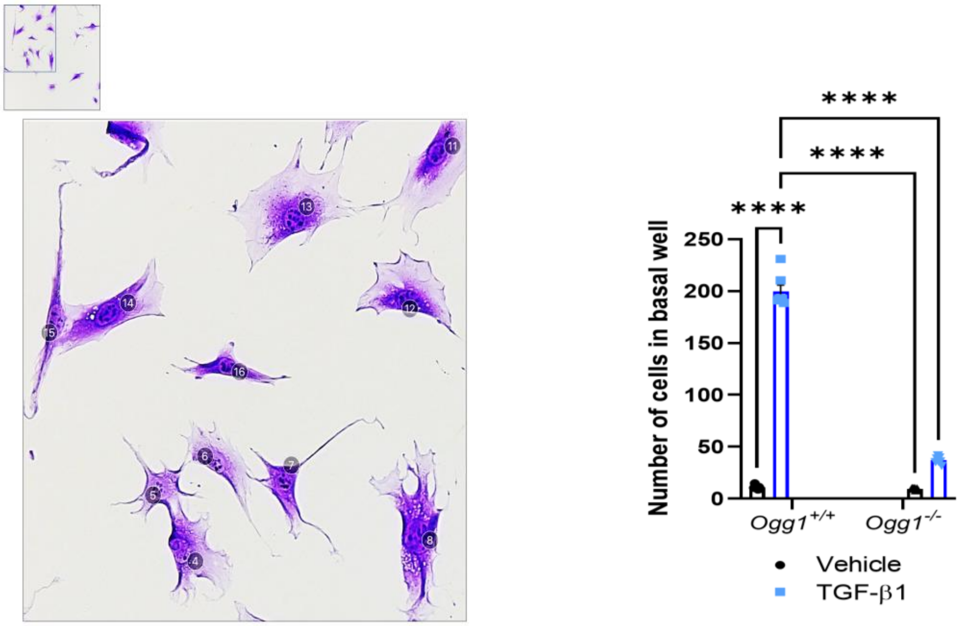

Supplementary Figure 22: Representative automated cell counting for transwell assay of OGG1 proficient MF cells following crystal violet staining. Migration of *Ogg1*$^{+/+}$ and *Ogg1*$^{-/-}$ MF cells into the basal well measured post-TGF-β1 induction. *Ogg1*$^{-/-}$ MF cells were compared to *Ogg1*$^{+/+}$ MF cells using a one-way ANOVA followed by a Dunnett's post-hoc test: ****P<0.0001. Data is representative of 4 independent experiments containing 2 biological replicates (scale bar=100 µm). Data are presented as means ±SEM. Statistical analysis was conducted using a Student's t-test (P<0.0001).

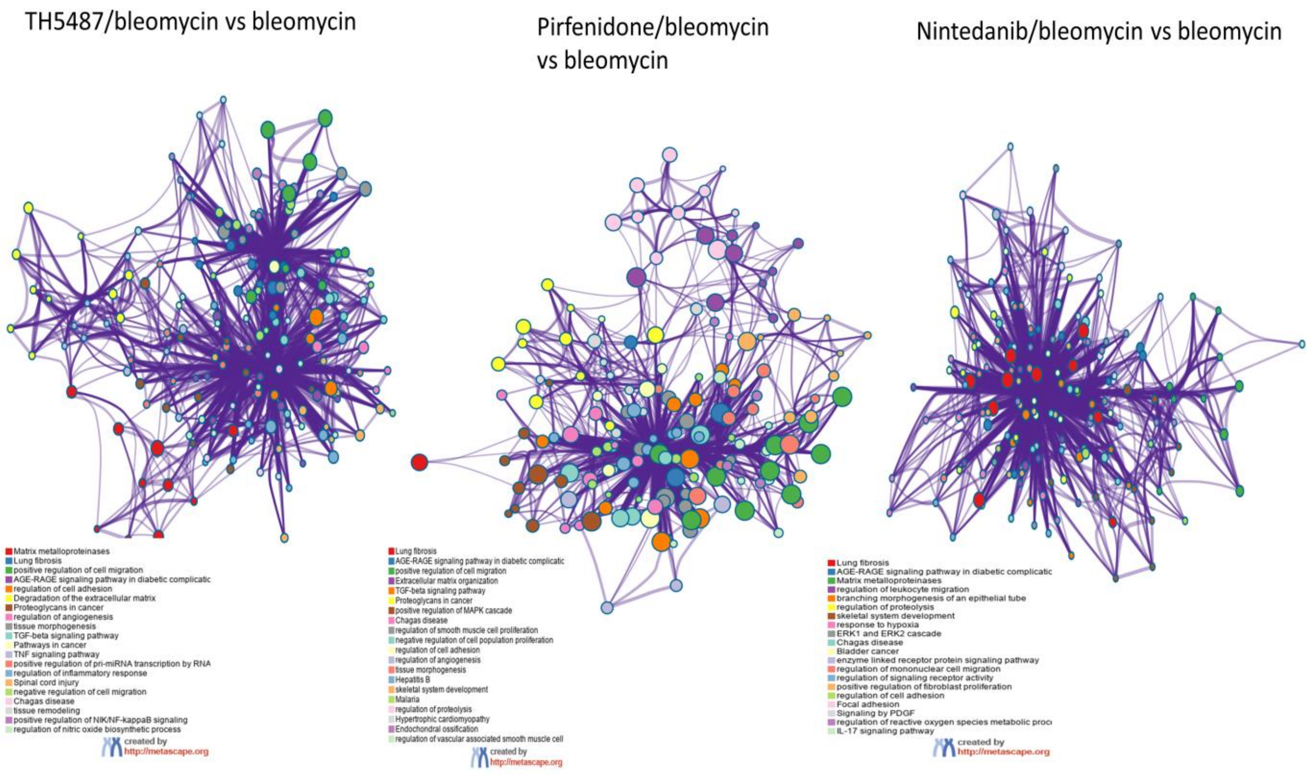

Supplementary Figure 23. Network of enriched terms following PCR array assay conducted on mouse lung tissue. Data are colored by cluster ID, where nodes that share the same cluster ID are typically close to each other (n=5 pooled samples per treatment condition).

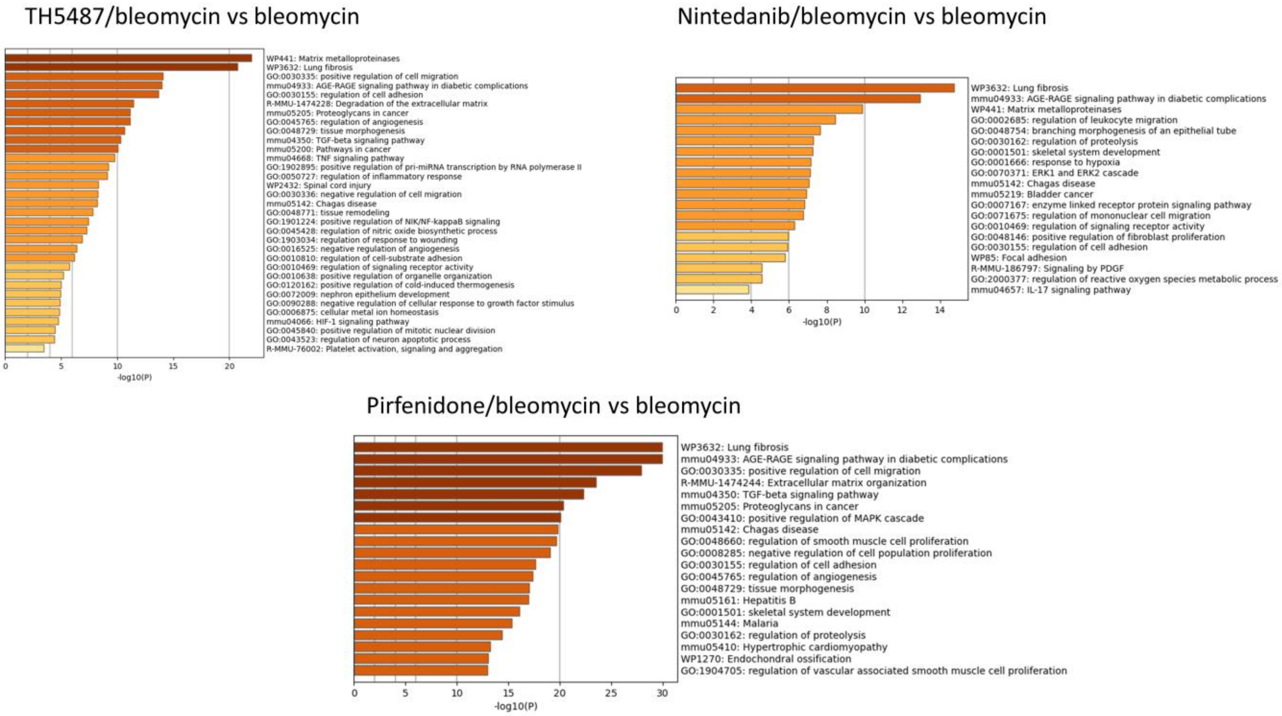

Supplementary Figure 24. Bar graph of enriched terms across input gene lists. Data are colored by *P*-values (n=5 pooled samples per treatment condition).

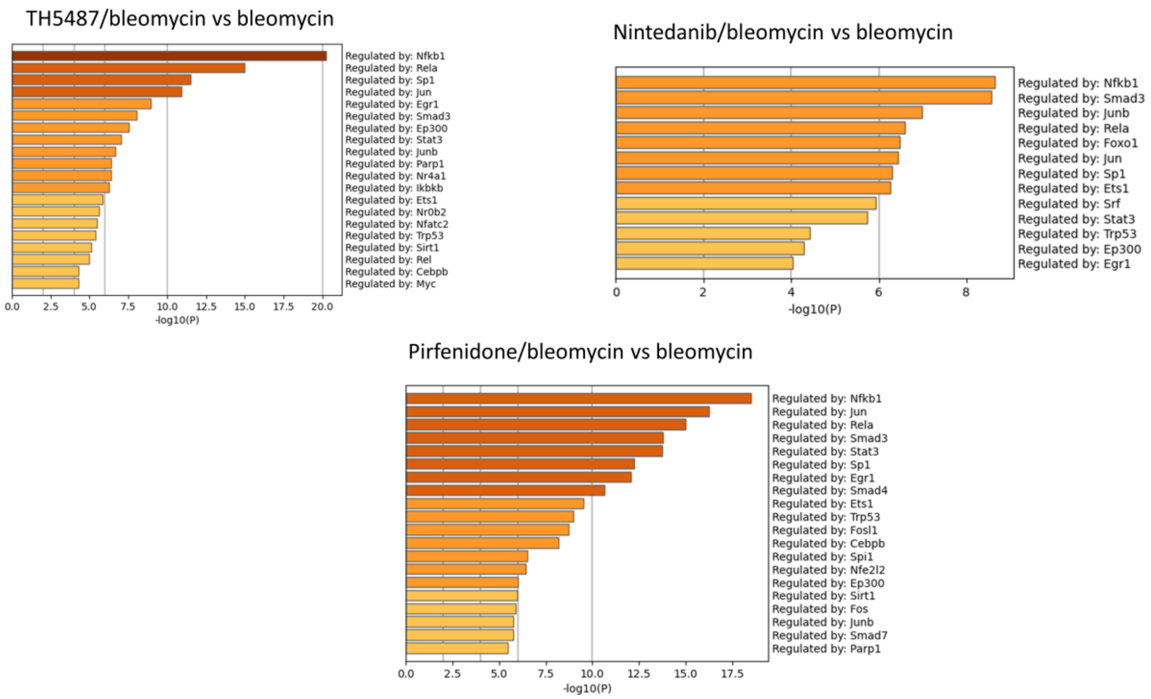

Supplementary Figure 25. Summary of enrichment analysis in transcriptional regulatory relationships. Data are unraveled by sentence-based text-mining (http://www.grnpedia.org/trrust;TRRUST). Data are colored by *P*-values (n=5 pooled samples per treatment condition).

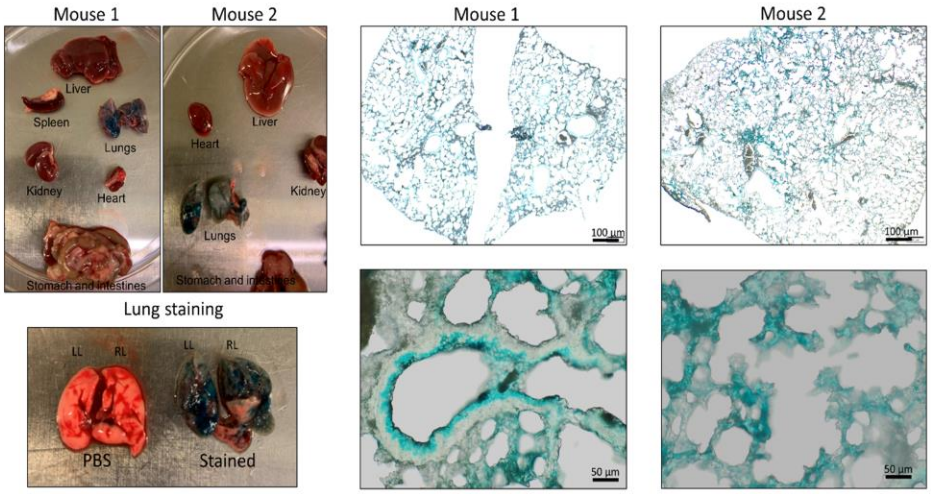

Supplementary Figure 26: **Murine lungs in two representative administrations of methylene blue solution.** Images allowed for the investigation of siRNA and bleomycin distribution within the mouse lungs. Results shown from 2 independent experiments.

Supplementary Table 1: Showing clinical data for patient samples included in this study. Healthy control samples were obtained from cancer patients undergoing lung resectioning (values not available-NA; F-female; M-male). FEV1- Forced expiratory volume in one second; FEV1%-percentage forced expiratory volume % of expected FEV1 (in relation to age, height, and sex); FEV1/FVC- represents the proportion of a person's vital capacity expired in the first second of forced expiration (FEV1) divided by the forced vital capacity (FVC); DLCO-diffusion capacity of the lungs to carbon monoxide (CO per unit time per mm of driving pressure of CO (cc of CO/sec/mm of Hg)).

**Patient ID**	**Sex**	**Diagnosis**	**FEV1**	**FEV1%**	**FVC**	**FEV1/FVC**	**DCLO (%)**
LURES 005	F	Adenocarcinoma	2.0	90	2.46	0.81	50
LURES 010	F	Atypical carcinoma	1.78	59	2.37	0.75	64
LURES 013	F	Adenocarcinoma	2.5	101	3.37	0.74	96

LURES 018	M	Squamous epithelial cell cancer	3.1	93	NA	NA	NA
LUEX 18	M	Lung Fibrosis	2.2	53	2.78	0.79	34
LUEX 25	M	Lung Fibrosis	1.22	41	1.56	0.81	NA
LUEX 55	M	Lung Fibrosis	2.2	73	2.52	0.87	26
LUEX 24	F	Lung Fibrosis	1.32	53	1.76	0.75	33

Supplementary Table 2. ARRIVE guidelines in the context of the current data set

ARRIVE guidelines		
Guideline	Criteria	Data in this manuscript
1.Study design	For each experiment, provide brief details of study design including: a. The groups being compared, including control groups. If no control group has been used, the rationale should be stated. b. The experimental unit (e.g. a single animal, litter, or cage of animals).	Data were compared to the bleomycin control group. Control groups were also included for vehicle administration, TH5487 only administration, anti-inflammatory response (Dexamethasone/bleomycin), and clinically-utilized drugs (pirfenidone and nintedanib).<br><br>The experimental unit in this case is the treatment group of mice included for each intervention.
2. Sample size	a. Specify the exact number of experimental units allocated to each group, and the total number in each experiment. Also indicate the total number of animals used. b. Explain how the sample size was decided. Provide details of any a priori sample size calculation, if done.	a. The following numbers of animals were allocated to each treatment and prophylactic group respectively, with the numbers representative of data included in the manuscript.<br><br>-Bleomycin (n=5 and n=13)<br>-Bleomycin/Ogg1 siRNA (n=5; n=0)<br>-PBS/Ogg1 siRNA (n=4; n=0)<br>-Vehicle (n=4; n=8)<br>-Bleomycin/TH5487 (n=8; n=13)<br>-Bleomycin/Pirfenidone (n=8; n=8)<br>-Bleomycin/Nintedanib (n=8; n=8)<br>-Bleomycin/Dexamethasone (n=0; n=9)<br><br>The total animals used amounted to n=148 and were allocated accordingly:

		-Bleomycin (n=13 and n=18)<br><br>-Bleomycin/Ogg1 siRNA (n=7; n=0)<br><br>-PBS/Ogg1 siRNA (n=4; n=0)<br><br>-Vehicle (n=4; n=8)<br><br>-PBS/TH5487 (n=0; n=8)<br><br>-Bleomycin/TH5487 (n=10; n=15)<br><br>-Bleomycin/Pirfenidone (n=10; n=13)<br><br>-Bleomycin/Nintedanib (n=10; n=13)<br><br>-Bleomycin/Dexamethasone (n=0; n=15)<br><br>b. The power calculations were determined *a priori* as follows (added to the reporting summary), which we believe to be statistically sound. From an initial pilot study involving 3 groups of mice (*N*=5 per group) we determined an initial effect size (f) of 0.6 allowing us to perform formal power and sample size calculations. Using the freely downloadable software G Power (Faul, Erdfelder, Lang and Buchner, 2007 https://pubmed.ncbi.nlm.nih.gov/17695343/), we calculated an *a priori* sample size based on the following values: α=0.05; Power=0.9; number of groups=5. Based on the pilot study data, an ANOVA with fixed effects, omnibus, one-way was used to calculate the ideal total sample size value of 50 (*N*=10). However, the study was designed with the knowledge that the administration of bleomycin causes mortality in 10-20% of the mice before the crucial onset of fibrosis. With this knowledge, the corrected group sizes for the bleomycin administered groups would be calculated at a minimum of *N*=12. To account for unexpected murine death (due to intraperitoneal injection) we allowed for *N*=13 mice per bleomycin treated group. The remaining group, namely vehicle only was decided upon considering the 3 R's in the ARRIVE guidelines for animal use. It was decided that with no changes expected within the vehicle only group, based on our preliminary studies, a smaller group size could be used. This approach was sufficient to detect significant biological differences and offer translational value from this set of experiments.

3. Inclusion and exclusion criteria	a. Describe any criteria used for including and excluding animals (or experimental units) during the experiment, and data points during the analysis. Specify if these criteria were established a priori. If no criteria were set, state this explicitly. b. For each experimental group, report any animals, experimental units or data points not included in the analysis and explain why. If there were no exclusions, state so. c. For each analysis, report the exact value of n in each experimental group.	a. No data were excluded from the analysis but animals that did not survive to Day 15 of the model were not processed for further readouts.<br><br>-Bleomycin (n=5 and n=13)<br>-Bleomycin/Ogg1 siRNA (n=5; n=0)<br>-PBS/Ogg1 siRNA (n=4; n=0)<br>-Vehicle (n=4; n=8)<br>PBS/TH5487 (n=0; n=8)<br>-Bleomycin/TH5487 (n=8; n=13)<br>-Bleomycin/Pirfenidone (n=8; n=8)<br>-Bleomycin/Nintedanib (n=8; n=8)<br>-Bleomycin/Dexamethasone (n=0; n=9)
4. Randomization	a. State whether randomisation was used to allocate experimental units to control and treatment groups. If done, provide the method used to generate the randomisation sequence. b. Describe the strategy used to minimise potential confounders such as the order of treatments and measurements, or animal/cage location. If	a. Mice were randomly assigned to each group using a simple randomization approach.<br>b. Confounders were reduced by randomizing treatment administration. Mice were also euthanized in groups based on a randomized order. The Excel function =INT(RAND()*group number) was used to calculate which groups would be treated or euthanized.

	confounders were not controlled, state this explicitly.	
5. Blinding	Describe who was aware of the group allocation at the different stages of the experiment (during the allocation, the conduct of the experiment, the outcome assessment, and the data analysis).	Two of the investigators (JB/LT) were aware of which animals needed to be dosed with TH5487/bleomycin and as such were not truly blinded to the outcome of the study. However, investigators not involved in the animal experimentation (RB) were blinded to the outcome and conducted downstream analyses in an unbiased manner. Therefore, we have not biased the study in terms of any of the results, but it is not correct to state that complete blinding was used.
6. Outcome measures	a. Clearly define all outcome measures assessed (e.g. cell death, molecular markers, or behavioral changes). b. For hypothesis-testing studies, specify the primary outcome measure, i.e. the outcome measure that was used to determine the sample size	a. Outcome measures in this study were mainly derived post-euthanization, with the exception of murine weight. End point-measurements included but were not limited to, lung/BALF inflammatory cell recruitment, cytokine production, collagen deposition, profibrotic gene regulation, profibrotic protein production, and histological damage.<br>b. During the sample size calculation a pilot study was conducted and hydroxyproline measurements were used as the outcome measure for comparison.
7. Statistical methods	a. Provide details of the statistical methods used for each analysis, including software used. b. Describe any methods used to assess whether the data met the assumptions of the statistical approach, and what was done if the assumptions were not met.	In this study, groups of three or more mice were compared using one-way analysis of variance (ANOVA) with Dunnett's post hoc test. In experiments using two groups, results were compared using unpaired *t* test with Welch's correction. Results in this study are displayed throughout as mean ± SEM. Statistical testing was carried out using GraphPad Prism 9.1.1 (San Diego, USA) with statistical significance defined as $P < 0.05$.
8. Experimental animals	a. Provide species-appropriate details of the animals used, including species,	10-12-week-old male C57Bl/6 mice (Janvier, Le Genest-Saint-Isle, France) were housed at least 2 weeks in the animal facility at the Biomedical Service Division at Lund University before initiating

	strain and sub strain, sex, age or developmental stage, and, if relevant, weight. b. Provide further relevant information on the provenance of animals, health/immune status, genetic modification status, genotype, and any previous procedures.	experiments and were provided with food and water *ad libitum* throughout the study.
9. Experimental procedures	For each experimental group, including controls, describe the procedures in enough detail to allow others to replicate them, including: a. What was done, how it was done and what was used. b. When and how often. c. Where (including detail of any acclimatization periods). d. Why (provide rationale for procedures).	10-12-week-old male C57Bl/6 mice (Janvier, Le Genest-Saint-Isle, France) were housed at least 2 weeks in the animal facility at the Biomedical Service Division at Lund University before initiating experiments and were provided with food and water *ad libitum* throughout the study. In addition, mice underwent the following procedures:<br><br>-Bleomycin administration: Bleomycin (i.t.; 2.5 U/kg) or saline control was administered once using a microsprayer device under anesthesia (isofluorane).<br><br>-Drug/siRNA treatment: Compounds were administered at different time points in the two different models with mice in the treatment group receiving treatment from Day 15 onwards and mice in the prophylactic group receiving treatment 1h after bleomycin administration.<br><br>The treatment for each group is outlined below as follows:<br><br>-Bleomycin: Mice received bleomycin (2.5 U/kg; i.t.) via microsprayer. Mice were subsequently administered vehicle (150 uL i.p.; once daily) at day 15-21 or day 1-21.

		-Bleomycin/Ogg1 siRNA:<br><br>Mice received bleomycin (2.5 U/kg; i.t.) via microsprayer. Mice were subsequently administered siRNA (25 µg i.t.; once) at day 15.<br><br>PBS/Ogg1 siRNA:<br><br>Mice received saline (50 uL; i.t.) via microsprayer. Mice were subsequently administered siRNA (50 µg i.t.; once) at day 15.<br><br>-Vehicle:<br><br>Mice received saline (50 uL; i.t.) via microsprayer. Mice were subsequently administered vehicle (150 uL i.p.; once daily) at day 15-21.<br><br>-PBS/TH5487:<br><br>Mice received saline (50 uL; i.t.) via microsprayer. Mice were subsequently administered TH5487 (40 mg/kg i.p.; once daily) at day 15-21.<br><br>-Bleomycin/TH5487:<br><br>Mice received bleomycin (2.5 U/kg; i.t.) via microsprayer. Mice were subsequently administered TH5487 (40 mg/kg i.p.; once daily) at day 15-21 or day 1-21.<br><br>-Bleomycin/Pirfenidone:<br><br>Mice received bleomycin (2.5 U/kg; i.t.) via microsprayer. Mice were subsequently administered pirfenidone (300 mg/kg p.o.; once daily) at day 15-21 or day 1-21.<br><br>-Bleomycin/Nintedanib:<br><br>Mice received bleomycin (2.5 U/kg; i.t.) via microsprayer. Mice were subsequently administered

		nintedanib (60 mg/kg p.o.; once daily) at day 15-21 or day 1-21.<br><br>-Bleomycin/Dexamethasone:<br>Mice received bleomycin (2.5 U/kg; i.t.) via microsprayer. Mice were subsequently administered dexamethasone (10 mg/kg i.p.; once daily) at day 15-21 or day 1-21.
10. Results	For each experiment conducted, including independent replications, report: a. Summary/descriptive statistics for each experimental group, with a measure of variability where applicable (e.g. mean and SD, or median and range). b. If applicable, the effect size with a confidence interval.	a. Each readout contains a description of the statistical method used (with appropriate P value, with results reported as means and SD's.<br>b. Effect sizes have not been provided
11. Abstract	Provide an accurate summary of the research objectives, animal species, strain and sex, key methods, principal findings, and study conclusions	All relevant experimental details are provided in the abstract, with key methods, findings and conclusions all highlighted.
12. Background	a. Include sufficient scientific background to understand the rationale and context for the study, and explain	a. The background to the study is supportive and explains the context of the research.<br>b. The reasons for using the bleomycin model have been highlighted 'This model reproduces several phenotypic features of human IPF, including peripheral alveolar septal thickening, dysregulated

	the experimental approach. b. Explain how the animal species and model used address the scientific objectives and, where appropriate, the relevance to human biology	cytokine production, and immune cell influx resulting in fibrosis.’
13. Objectives	Clearly describe the research question, research objectives and, where appropriate, specific hypotheses being tested.	The goal of this study was to test a novel pharmaceutical approach to inhibit OGG1, ultimately leading to the inhibition of fibrosis-related progression. Initial *in vitro* experiments in bronchial epithelial and fibroblast cells displayed decreased fibrosis-related phenotypic features. Subsequent *in vivo* murine studies using intratracheally-administered bleomycin were chosen as well-established and relevant models of experimental lung fibrosis.
14. Ethical statement	Provide the name of the ethical review committee or equivalent that has approved the use of animals in this study, and any relevant licence or protocol numbers (if applicable). If ethical approval was not sought or granted, provide a justification.	All animal experiments were approved by the Malmö-Lund Animal Care Ethics Committee (M17009-18). Human lung tissue was obtained after written informed consent, approval by the Regional Ethical Review Board in Lund (approval no. LU412-03) and performed in accordance with the Declaration of Helsinki as well as relevant guidelines and regulations.
15. Housing and husbandry	Provide details of housing and husbandry conditions, including any environmental enrichment.	10-12-week-old male C57Bl/6 mice (Janvier, Le Genest-Saint-Isle, France) were housed at least 2 weeks in the animal facility at the Biomedical Service Division at Lund University before initiating experiments and were provided with food and water *ad libitum* throughout the study. Mice were provided with environmental enrichment which included cardboard tubes, chew sticks, and shredded paper.
16. Animal care and monitoring	a. Describe any interventions or steps taken in the experimental protocols to reduce pain, suffering and	Mice were anaesthetized during all invasive procedures (i.t. administration). In addition, the mice were euthanized as soon as the weight crossed the 20% cutoff mark or if the mice displayed any signs of distress as listed in our ethical permit. The general humane endpoint is indicated by many signs

	distress. b. Report any expected or unexpected adverse events. c. Describe the humane endpoints established for the study, the signs that were monitored and the frequency of monitoring. If the study did not have humane endpoints, state this.	including a loss of 20% of original mouse weight and changes in mouse behavior including (amended in revised manuscript):<br>- discomfort or stress: (e.g. dull/staring coat (hair erect); decreased or increased activity; avoidance behaviour; isolation from the group; depressed; decreased appetite)<br>- deterioration (e.g. Discharge from eyes; loss of general condition; anorexia; dehydration (tented skin/sunken eyes); weakness; decreased motility)<br>- distress: (e.g. very weak; unresponsive to touch; unconscious; convulsing; difficulty breathing)<br>- If obvious distress (bleeding trachea, labored breathing etc.) following intratracheal administration of compound/bleomycin is seen, the mice will be immediately euthanized.<br>- Mice that experience severe adverse reactions to the test compound or anaesthetic drug and are deemed to be in distress will be euthanised. Animals showing neurological signs, that have convulsions, demonstrate severe weakness, avoidance behaviour, display signs of depression, show respiratory distress, or open-mouthed breathing will humanely be euthanized prior to the end point.
17. Interpretation	a. Interpret the results, taking into account the study objectives and hypotheses, current theory and other relevant studies in the literature. b. Comment on the study limitations including potential sources of bias, limitations of the animal model, and	Together, our findings demonstrate that TH5487 possesses a mechanism of action targeting OGG1 to suppress IPF, which is distinct from currently employed therapeutic interventions. This study further elucidates the downstream effects of this approach, decreasing myofibroblast transition, fibroblast migration, inflammatory cell recruitment, and eventual inhibition of fibrotic-related lung remodeling. These data show promising therapeutic effects of TH5487 in a mouse model of IPF, motivating further pre-clinical evaluation.<br>Important limitations addressed in this study include, whether treatment using TH5487 can

	imprecision associated with the results.	display utility in human IPF pathologies. Whilst the translational aspect of the study is suggested using human lung sections, it is important to demonstrate that TH5487 treatment successfully decreased OGG1 levels and subsequent IPF lung damage in human clinical trials. Additional limitations in this study include the lack of monitoring of potential off-target effects induced by targeting OGG1. Whilst no obvious reductions in key murine health status measures were observed in this or other studies, any small-molecule utilization should be accompanied by long term monitoring of adverse effects to ensure safe therapeutic usage. Furthermore, *Ogg1*-/- mice display no deleterious pathological changes, suggesting specific *Ogg1* targeting may be safe. In any case, further efforts are required before progressing this compound to clinical trials.
18. Generalisability/translation	Comment on whether, and how, the findings of this study are likely to generalise to other species or experimental conditions, including any relevance to human biology (where appropriate).	Our data demonstrate targeting *Ogg1* suppresses TGF-β1 and several key immune modulatory cytokines and chemokines in murine BALF, lungs, and plasma. Treatment with both TH5487 or *Ogg1* siRNA resulted in decreased profibrotic cytokine expression and diminished immune cell recruitment to the lung.<br><br>These findings are relevant for numerous diseases with an inflammatory related description.
19. Protocol registration	Provide a statement indicating whether a protocol (including the research question, key design features, and analysis plan) was prepared before the study, and if and where this protocol was registered.	The study plan was included as part of several grant applications which have been listed in the manuscript. The work was supported by grants from:<br>Swedish Research Council 2020-011166 (AE)<br>The Swedish Heart and Lung Foundation 20190160 (AE)<br>The Swedish Government Funds for Clinical Research 46402 (ALF; AE)<br>The Alfred Österlund Foundation (AE)<br>Vinnova Swelife 2, 2018-03232 (AE, TH, CK)<br>Horizon 2020 ERC-PoC (TH)<br>US NIH, National Institute of Allergy and Infectious Diseases, AI062885 (IB)

20. Data access	Provide a statement describing if and where study data are available.	Mass spectrometry proteomics data and initial search results have been deposited to the ProteomeXchange Consortium (http://proteomecentral.proteomexchange.org) via the PRIDE partner repository [1] with the dataset identifier PXD029625 (Username: reviewer_pxd029625@ebi.ac.uk ; Password: 5YIGmBWu; will be made public upon acceptance of the manuscript). Downstream analysis R code is available at https://github.com/heuselm/DiffTestR/tree/Tanner2021. All remaining data are available in the main text or the supplementary materials.
21. Declaration of interests	a. Declare any potential conflicts of interest, including financial and non-financial. If none exist, this should be stated. b. List all funding sources (including grant identifier) and the role of the funder(s) in the design, analysis and reporting of the study	T.H. is listed as inventor on a provisional U.S. patent application no. 62/636983, covering OGG1 inhibitors. The patent is fully owned by a nonprofit public foundation, the Helleday Foundation, and T.H. is a member of the foundation board developing OGG1 inhibitors toward the clinic. An inventor reward scheme is under discussion. The remaining authors declare no competing financial interests.<br><br>The work was supported by grants from:<br>Swedish Research Council 2020-011166 (AE)<br>The Swedish Heart and Lung Foundation 20190160 (AE)<br>The Swedish Government Funds for Clinical Research 46402 (ALF; AE)<br>The Alfred Österlund Foundation (AE)<br>Vinnova Swelife 2, 2018-03232 (AE, TH, CK)<br>Horizon 2020 ERC-PoC (TH)<br>US NIH, National Institute of Allergy and Infectious Diseases, AI062885 (IB)<br><br>Author contributions:<br>Conceptualization: AE, LT, ABS, CK, JB, IB<br>Methodology: LT, ABS, RKVB, MH, TM, CAQK, JM, RMO, CC, CKA, JSE, OW, TH, JB, IB<br>MS data management & analysis: MH

		Investigation: LT, ABS, RKVB, MH, TM, CAQK, RMO, CC, CKA, JSE, JB, IB<br><br>Funding acquisition: AE, CK, TH<br><br>Project administration: AE, CK, TH<br><br>Supervision: AE, CK, TH<br><br>Writing – original draft: LT<br><br>Writing – review & editing: LT, ABS, AE, CK, MH, JB, IB

Full blots for Supplementary materials:

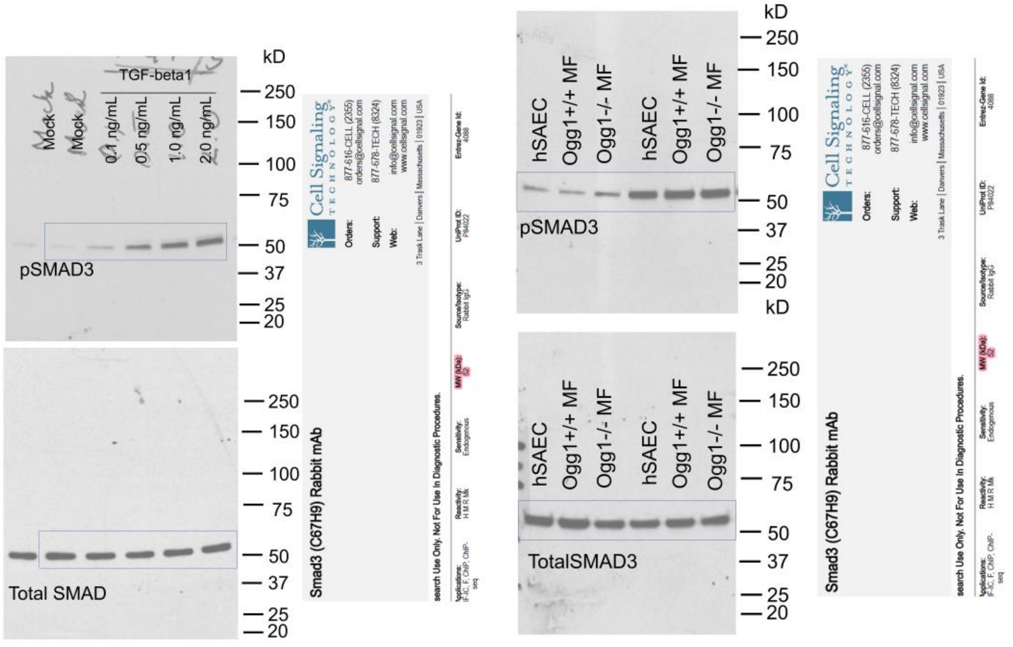

Supp. figure 1A and B blots: Uncropped blots for Supp. Fig. 1A and B. Samples were assessed for phosphorylated SMAD3 (pSMAD3) and Total SMAD3. Results shown from 3 independent experiments.
