# Supplementary material for: Small-molecule-mediated OGG1 inhibition attenuates pulmonary inflammation and lung fibrosis in a murine lung fibrosis model
Source: Nat Commun. 2023 Feb 6;14:643. doi: 10.1038/s41467-023-36314-5 (PMC9902543; doi:10.1038/s41467-023-36314-5)
Supplement: Supplementary file 3 — Reporting Summary [file 41467_2023_36314_MOESM3_ESM.pdf]

nature portfolio

Corresponding author(s): Lloyd Tanner

Last updated by author(s): Dec 10, 2022

Reporting Summary

Nature Portfolio wishes to improve the reproducibility of the work that we publish. This form provides structure for consistency and transparency in reporting. For further information on Nature Portfolio policies, see our Editorial Policies and the Editorial Policy Checklist.

Statistics

For all statistical analyses, confirm that the following items are present in the figure legend, table legend, main text, or Methods section.

n/a	Confirmed	
☐	☒	The exact sample size (n) for each experimental group/condition, given as a discrete number and unit of measurement
☐	☒	A statement on whether measurements were taken from distinct samples or whether the same sample was measured repeatedly
☐	☒	The statistical test(s) used AND whether they are one- or two-sided<br>*Only common tests should be described solely by name; describe more complex techniques in the Methods section.*
☐	☒	A description of all covariates tested
☐	☒	A description of any assumptions or corrections, such as tests of normality and adjustment for multiple comparisons
☐	☒	A full description of the statistical parameters including central tendency (e.g. means) or other basic estimates (e.g. regression coefficient) AND variation (e.g. standard deviation) or associated estimates of uncertainty (e.g. confidence intervals)
☐	☒	For null hypothesis testing, the test statistic (e.g. F, t, r) with confidence intervals, effect sizes, degrees of freedom and P value noted<br>*Give P values as exact values whenever suitable.*
☒	☐	For Bayesian analysis, information on the choice of priors and Markov chain Monte Carlo settings
☒	☐	For hierarchical and complex designs, identification of the appropriate level for tests and full reporting of outcomes
☒	☐	Estimates of effect sizes (e.g. Cohen's d, Pearson's r), indicating how they were calculated

Our web collection on statistics for biologists contains articles on many of the points above.

Software and code

Policy information about availability of computer code

Data collection	BD Accuri C6 Plus Software (v 1.0.27.1) was used for flow cytometry data collection. Sample sizes were calculated by power analysis (G Power Software). CellSens Entry software (Olympus, Tokyo, Japan). Fluorescence was quantified using ImageJ software (Java 1.8.0_172). Bio-Plex Manager 6.2 software (Bio-Rad, Richmond, CA).
Data analysis	Bio-Plex Manager software (v 6.2), Aperio CS2 image capture software (v 12.3), ImageJ software (Java 1.8.0_172), DIANN Toolset (v 1.7.12)-with packages including data.table, pheatmap, fviz, ggplot2, ggrepel and limma, R version 4.0.2, PANTHER classification system (v 15.0), GraphPad Prism (v 9.1.1). Downstream analysis R code is available at https://github.com/heuselm/DiffTestR/tree/Tanner2021.

For manuscripts utilizing custom algorithms or software that are central to the research but not yet described in published literature, software must be made available to editors and reviewers. We strongly encourage code deposition in a community repository (e.g. GitHub). See the Nature Portfolio guidelines for submitting code & software for further information.

Data

Policy information about availability of data

All manuscripts must include a data availability statement. This statement should provide the following information, where applicable:

- Accession codes, unique identifiers, or web links for publicly available datasets
- A description of any restrictions on data availability
- For clinical datasets or third party data, please ensure that the statement adheres to our policy

Mass spectrometry proteomics data and initial search results have been deposited to the ProteomeXchange Consortium (http://proteomecentral.proteomexchange.org) via the PRIDE partner repository [1] with the dataset identifier PXD029625 (Username: reviewer_pxd029625@ebi.ac.uk ; Password: 5YIGmBWu; will be made public upon acceptance of the manuscript). Source data and all relevant raw data are provided with this paper.

Human research participants

Policy information about studies involving human research participants and Sex and Gender in Research.

Reporting on sex and gender	IPF is a disease mainly affecting the male population~70% prevalence (PMID: 34422868). We have therefore included 3 male and 1 female IPF patient to represent this in our study.
Population characteristics	Patients were selected following HRCT positivity (opaque fibrosis-like areas and honeycombing in the lung parenchyma). Patients were also older than 55 and predominantly male given the disease prevalence. Control patients were also older than 50 and had undergone explant surgery in relation to lung transplantation.
Recruitment	Macroscopically normal, tumor-free lung tissue samples were obtained during transplantation or resection from patients undergoing cancer surgery/IPF lung resection.
Ethics oversight	Human lung tissue was obtained after written informed consent, approval by the Regional Ethical Review Board in Lund (approval no. LU412-03) and performed in accordance with the Declaration of Helsinki as well as relevant guidelines and regulations.

Note that full information on the approval of the study protocol must also be provided in the manuscript.

Field-specific reporting

Please select the one below that is the best fit for your research. If you are not sure, read the appropriate sections before making your selection.

☒ Life sciences ☐ Behavioural & social sciences ☐ Ecological, evolutionary & environmental sciences

For a reference copy of the document with all sections, see nature.com/documents/nr-reporting-summary-flat.pdf

Life sciences study design

All studies must disclose on these points even when the disclosure is negative.

Sample size	Sample sizes were chosen based on previous publications (PMID: 35013220, 28230051 and 31666402) with number of replicates include in each figure legend. In order to ensure robust statistical analysis, a sample size of n=4 or greater was used throughout the study. All experiments were completed in biological duplicates or greater. Murine experiments utilized n=6 per group or greater, with murine numbers based on historical data and assumed power calculations and effect size.
Data exclusions	No animal or samples data were excluded unless the animal died prior to 14 days.
Replication	Experiments were performed successfully, with results obtained from 2 or more biological replicates.
Randomization	Mice were randomly allocated into each group prior to commencement of studies. Cell lines were randomly seeded into different wells prior to treatment. For human tissue samples, IPF and control tissues were randomly selected from a biobank with the only sample selection criteria involving age-matching samples.
Blinding	Investigators were blinded to group allocation during all data collection and analysis.

Reporting for specific materials, systems and methods

We require information from authors about some types of materials, experimental systems and methods used in many studies. Here, indicate whether each material, system or method listed is relevant to your study. If you are not sure if a list item applies to your research, read the appropriate section before selecting a response.

Materials & experimental systems

n/a	Involved in the study
	[x] Antibodies
	[x] Eukaryotic cell lines
[x]	Palaeontology and archaeology
	[x] Animals and other organisms
[x]	Clinical data
[x]	Dual use research of concern

Methods

n/a	Involved in the study
[x]	ChIP-seq
	[x] Flow cytometry
[x]	MRI-based neuroimaging

Antibodies

Antibodies used

The following antibodies were used during this study: APC Rat anti-CD11b (BD 553312; 1:200), PE CY 7 Rat anti-CD11c (BD 558 079; 1:200), PE Rat anti-LY6G (BD 551461; 1:200), PE CY7 Rat anti-SiglecF (BD 562680 (1:200), PE Rat anti-I-Ad/I-Ed (BD 558593; 1:200). Rabbit anti-fibronectin (ab268020; 1:500), Rabbit anti-LY6G (ab238132; 1:1000), Mouse anti-COL1A1 (ab88147; 1:200), Rabbit anti-myeloperoxidase (ab208670; 1:200), Rabbit anti-mannose receptor (ab64693; 1:500), Rat anti-f4/80 (ab6640; 1:500), Rabbit anti-MADH7/SMAD7 (ab216428; 1:500), Rabbit anti-alpha smooth muscle actin (ab5694; 1:300), Rabbit anti-OGG1 (PA1-31402; 1:500), Rabbit anti-vimentin (ab92547; 1:300), Mouse anti-GAPDH (ab8245; 1:1000), Alexa fluor-conjugated (488) goat anti-mouse secondary (ab150113; 1:2000), Alexa fluor-conjugated (647)
goat anti-rabbit secondary (ab150083; 1:1000).

Validation

All primary and secondary antibodies were purchased from commercial vendors and have been utilized in multiple publications. APC Rat anti-CD11b- https://www.bdbiosciences.com/en-se/products/reagents/flow-cytometry-reagents/research-reagents/single-color-antibodies-ruo/purified-rat-anti-cd11b.557394
PE CY 7 Hamster anti-CD11c- https://www.bdbiosciences.com/en-eu/products/reagents/flow-cytometry-reagents/research-reagents/single-color-antibodies-ruo/pe-cy-7-hamster-anti-mouse-cd11c.561022
PE Rat anti-LY6G- https://www.bdbiosciences.com/en-ca/products/reagents/flow-cytometry-reagents/research-reagents/single-color-antibodies-ruo/pe-rat-anti-mouse-ly-6g.551461
PE CY7 Rat anti-SiglecF- https://www.bdbiosciences.com/en-eu/products/reagents/flow-cytometry-reagents/research-reagents/single-color-antibodies-ruo/pe-rat-anti-mouse-siglec-f.552126
PE Rat anti-I-Ad/I-Ed- https://www.bdbiosciences.com/en-se/products/reagents/flow-cytometry-reagents/research-reagents/single-color-antibodies-ruo/pe-rat-anti-mouse-i-ad-i-ed.558593
Rabbit anti-fibronectin- https://www.abcam.com/fibronectin-antibody-epr23110-46-ab268020.html
Rabbit anti-LY6G-https://www.abcam.com/ly6g-antibody-epr22909-135-ab238132.html
Mouse anti-COL1A1- https://www.abcam.com/collagen-i-antibody-3g3-bsa-and-azide-free-ab88147.html
Rabbit anti-myeloperoxidase- https://www.abcam.com/myeloperoxidase-antibody-epr20257-ab208670.html
Rabbit anti-mannose receptor- https://www.abcam.com/mannose-receptor-antibody-ab64693.html
Rat anti-f4/80- https://www.abcam.com/f480-antibody-cia3-1-macrophage-marker-ab6640.html
Rabbit anti-alpha smooth muscle actin- https://www.abcam.com/alpha-smooth-muscle-actin-antibody-ab5694.html
Rabbit anti-MADH7/SMAD7- https://www.abcam.com/madh7smad7-antibody-ab216428.html
Rabbit anti-OGG1- https://www.thermofisher.com/antibody/product/OGG1-Antibody-Polyclonal/PA1-31402
Rabbit-anti-vimentin-https://www.abcam.com/vimentin-antibody-epr3776-cytoskeleton-marker-ab92547.html
Mouse anti-GAPDH- https://www.abcam.com/gapdh-antibody-6c5-loading-control-ab8245.html
Alexa fluor-conjugated (488) goat anti-mouse secondary- https://www.abcam.com/goat-mouse-igg-hl-alexa-fluor-488-ab150113.html
Alexa fluor-conjugated (647) goat anti-rabbit secondary- https://www.abcam.com/goat-rabbit-igg-hl-alexa-fluor-647-preadsorbed-ab150083.html

Eukaryotic cell lines

Policy information about cell lines and Sex and Gender in Research

Cell line source(s)	pHLF (Lonza Biosciences), hSAEC (Lonza Biosciences), MEF (BNCC100518, ATCC), PC3 cells (CRL-1435™, ATCC), HaCat cells (AddexBio T0020001).
Authentication	These cell lines were purchased from commercial vendors (Lonza, ATCC, and AddexBio) and have been authenticated.
Mycoplasma contamination	All cell lines tested negative for mycoplasma contamination.
Commonly misidentified lines (See ICLAC register)	No commonly misidentified cell lines were used.

Animals and other research organisms

Policy information about studies involving animals; ARRIVE guidelines recommended for reporting animal research, and Sex and Gender in Research

Laboratory animals	10-12-week-old male C57Bl/6 mice (Janvier, Le Genest-Saint-Isle, France) were housed at least 2 weeks in the animal facility at the Biomedical Service Division at Lund University before initiating experiments and were provided with food (RM1 (P) 801151; SDS, Essex, UK) and water ad libitum throughout the study. Mice were housed within a facility utilizing 12h light/dark cycles, with temperatures maintained between 23 and 25 degrees celsius (humidity 50%).
Wild animals	No wild animals were used.
Reporting on sex	Male mice are commonly used in fibrosis studies due to the development of fibrosis and higher disease prevalence in the male human population.
Field-collected samples	No field collected samples were used.
Ethics oversight	All animal experiments were approved by the Malmö-Lund Animal Care Ethics Committee (M17009-18).

Note that full information on the approval of the study protocol must also be provided in the manuscript.

Flow Cytometry

Plots

Confirm that:

- [x] The axis labels state the marker and fluorochrome used (e.g. CD4-FITC).
- [x] The axis scales are clearly visible. Include numbers along axes only for bottom left plot of group (a 'group' is an analysis of identical markers).
- [x] All plots are contour plots with outliers or pseudocolor plots.
- [x] A numerical value for number of cells or percentage (with statistics) is provided.

Methodology

Sample preparation	BALF samples were obtained from mice and kept on ice, cells were spun down and stained followed by the addition of Lyse/Fix solution (BD 558049).
Instrument	BD Accuri C6 Plus (BD Biosciences, San Diego, CA, USA)
Software	Data were analyzed by BD Accuri C6 Plus Software (as indicated above).
Cell population abundance	No post-sort fractions were obtained
Gating strategy	We applied FSC and SSC parameters to exclude cell debris and doublets, with Live/dead staining carried out on 'total cell' gate. Neutrophils were identified as Ly6G+SSC+CD11b+<br>with inflammatory (Ly6G-SSC+CD11b+CD11c low) and alveolar macrophage (Ly6G-SSC+CD11b+CD11c high) populations defined accordingly.

- [x] Tick this box to confirm that a figure exemplifying the gating strategy is provided in the Supplementary Information.
